# Temporal loss of *En1* during limb development causes distinct phenotypes

**DOI:** 10.1101/gad.353542.125

**Published:** 2026-05-01

**Authors:** Alessa R. Ringel, Natalia Benetti, Andreas Magg, Fabian Groll, Robert Schöpflin, Mira Kühnlein, Asita Carola Stiege, Ute Fischer, Lars Wittler, Laurence Game, Stephan Lorenz, George Young, Stefan Mundlos, Lila Allou

**Affiliations:** 1 Research Group Development and Disease, Max-Planck Institute for Molecular Genetics, Berlin 14195, Germany;; 2 Institute for Medical and Human Genetics, Charité-Universitätsmedizin Berlin, Berlin 13353, Germany;; 3 Berlin-Brandenburg Center for Regenerative Therapies, Charité-Universitätsmedizin Berlin, Berlin 13353, Germany;; 4 Medical Research Council Laboratory of Medical Sciences (MRC LMS), London W12 0HS, United Kingdom;; 5 Institute of Clinical Sciences, Faculty of Medicine, Imperial College London, London SW7 5NH, United Kingdom;; 6 Department of Computational Molecular Biology, Max-Planck Institute for Molecular Genetics, Berlin 14195, Germany;; 7 Department of Developmental Genetics, Max-Planck Institute for Molecular Genetics, Berlin 14195, Germany;; 8 Sequencing Core Facility, Max-Planck Institute for Molecular Genetics, Berlin 14195, Germany

**Keywords:** *Engrailed-1*, limb patterning, spatiotemporal gene expression, CREs, congenital malformations

## Abstract

The precise spatiotemporal regulation of developmental genes is required for proper organogenesis. *Engrailed-1* (*En1*) is essential for dorsal–ventral patterning during mouse limb development from embryonic day 9.5 (E9.5) to E11.5. Previously, we identified the long noncoding RNA locus *Maenli*, which drives limb-specific *En1* expression at E9.5. Here, we uncover two intergenic enhancer elements, *LSEE1*&*2*, that are essential for *En1* expression at E10.5 and E11.5. We demonstrate that *Maenli* and *LSEE1&2* coordinate two transcriptional waves of *En1* expression that are essential for establishing dorsal–ventral limb identity. The early wave, controlled by *Maenli*, is required for ventral ectoderm and apical ectodermal ridge specification, while the late wave, associated with *LSEE1&2*, is critical for dorsal–ventral ectoderm specification in mouse limbs. Loss of *Maenli* versus *LSEE1&2* results in distinct developmental phenotypes by altering the spatiotemporal expression of key genes governing axial patterning pathways. Our study underscores how temporal *cis*-regulatory elements modulate lineage specification and developmental patterning through shaping spatiotemporal gene function during development, representing potential candidates underpinning isolated subphenotypes.

## Abstract (precis)

In this study, Ringel et al. show that the expression of *Engrailed-1* (*En1*)—a transcription factor essential for developmental patterning in the murine limb—is controlled by a lncRNA (*Maenli*) and two intergenic *cis*-regulatory elements (*LSEE1&2*). *Maenli* and *LSEE1&2* coordinate two transcriptional waves of *En1* expression that define timed axial patterning and drive distinct developmental phenotypes when altered.

Healthy embryonic development relies on the precise expression of genes in space and time. Such spatiotemporal regulation of developmental genes is orchestrated by the action of *cis*-regulatory elements (CREs), including enhancers (de Laat and Duboule 2013) and long noncoding RNA (lncRNA) loci (Mattick et al. 2023; Ferrer and Dimitrova 2024). Enhancers are short, noncoding DNA sequences that regulate transcription rates from promoters and can be located at varying distances from their target genes (de Laat and Duboule 2013; Serebreni and Stark 2021). Most current genome-wide enhancer mapping strategies rely on transcription factor binding (e.g., EP300), enrichment for specific posttranslational histone modifications, and/or the presence of enhancer-associated RNAs (eRNAs) (Cao et al. 2025). Based on specific combinations of posttranslational histone tail modifications, enhancers are functionally grouped into inactive/neutral/primed enhancers marked by histone H3 lysine 4 monomethylation (H3K4me1), poised enhancers marked by both H3K4me1 and histone H3 lysine 27 trimethylation (H3K27me3), and active enhancers marked by H3K4me1 and histone H3 lysine 27 acetylation (H3K27ac) (Cao et al. 2025). However, this paradigm is being increasingly challenged. Recent studies have identified noncanonical enhancers that regulate gene expression and cell fate specification despite lacking typical marks associated with active enhancers such as H3K27ac and eRNAs (Cao et al. 2025). The interaction between enhancers and their target promoters is enabled by folding of the chromatin (Kagey et al. 2010; Furlong and Levine 2018), often constrained to topologically associated domains (TADs)—genomic regions with higher chromatin interaction frequencies within themselves than the rest of the genome (Robson et al. 2019; Allou and Mundlos 2023). In addition to enhancers, lncRNA loci have gained recognition in regulating tissue-specific developmental gene expression in recent years (Mattick et al. 2023; Ferrer and Dimitrova 2024). LncRNAs are noncoding transcripts >500 bp that are typically expressed at low levels compared with mRNAs (Mattick et al. 2023; Ferrer and Dimitrova 2024). Some lncRNA loci have been reported to function as CREs (Mattick et al. 2023; Ferrer and Dimitrova 2024).

Developmental genes are often regulated by a complex interplay of multiple CREs that can cooperate in an additive, synergistic, or redundant manner (van Arensbergen and van Steensel 2017; Osterwalder et al. 2018). Here, we dissected the regulatory architecture of the *Engrailed-1* (*En1*) gene during limb development by combining in vivo CRISPR/Cas9 genome editing, gene expression analyses, and enhancer reporter assays. *En1* encodes the homeobox transcription factor EN1, which can act as both a transcriptional activator and a repressor (Ma et al. 2024) and plays a key role during early embryogenesis. As such, its precise spatiotemporal expression is crucial for the development of several tissues like bone, muscle, cartilage, and skin (Ma et al. 2024). In mice, *En1* is first expressed at embryonic day 8 (E8) in the midbrain/hindbrain junction. By E9.5, it is found in the mesenchyme adjacent to the midbrain/hindbrain junction, the developing pituitary gland, the somites, and the limb buds. In the limbs, it is specifically expressed in the ventral ectoderm and apical ectodermal ridge (AER) (Ma et al. 2024). From E10.5 onward, *En1* expression extends to other structures, including the central nervous system, spinal cord, tail bud, and mandibular arch (Ma et al. 2024).

Homozygous *En1* knockout mice die shortly after birth and display multiple developmental abnormalities, including brain and skeletal (sternum, rib, and limb) defects (Wurst et al. 1994; Ma et al. 2024). The embryonic limb phenotype manifests as polydactyly, syndactyly, ectopic ventral digits and loss or incomplete formation of the falciform and sesamoid cartilages (Wurst et al. 1994; Ma et al. 2024). Deleting only the homeobox-coding sequence of *En1* allows rare survival of mice that exhibit a postnatal double dorsal limb phenotype. This phenotype consists of the dorsalization of ventral structures (ectopic ventral or circumferential claws, ectopic ventral hairs, loss of pads [nail-like differentiation], hyperpigmentation on the ventral epidermis, and digits flexed dorsally rather than ventrally) in addition to the embryonic limb phenotype described above (Loomis et al. 1996; Hanks et al. 1998). In this study, we used the polydactyly/syndactyly and ectopic ventral or circumferential nails to assess the severity of the embryonic and postnatal limb phenotypes, respectively.

The *En1* mutant limb phenotype is associated with molecular changes (Loomis et al. 1998). *En1* helps to establish the dorsal–ventral limb identity in part by restricting *Wnt7a* expression to the dorsal ectoderm. Loss of *En1* results in expanded *Wnt7a* expression into the ventral limb ectoderm, leading to abnormal ventral ectoderm specification at early stages (Loomis et al. 1998). Consequently, *Lmx1b*, a downstream target of Wnt7a, becomes misexpressed in the ventral mesenchyme at later stages (Loomis et al. 1998). Moreover, *En1* loss causes aberrant maturation of the AER, characterized by a proximoventral expansion of the *Fgf8* expression domain that gives rise to a secondary AER capable of promoting ectopic ventral digits on the proximal paws of some mutants (Loomis et al. 1998). Analysis of *En1*/*Wnt7a* double mutants demonstrated that the dorsalizing gene *Wnt7a* is required for the formation of the ectopic AERs in *En1* mutants (Loomis et al. 1998).

While the phenotypic consequences of *En1* loss in the developing limb have been well characterized (Wurst et al. 1994; Loomis et al. 1996; Hanks et al. 1998; Ma et al. 2024), less is known about the regulatory elements controlling *En1* expression. *En1* is positioned in an ∼650 kb TAD in which we previously identified the lncRNA locus *Maenli* (master activator of *engrailed-1* in the limb). *Maenli* initiates *En1* expression in the mouse limb bud at E9.5 (Allou et al. 2021). However, at E10.5 and E11.5, *Maenli* transcriptional activity significantly decreases, yet *En1* expression is maintained (Allou et al. 2021), suggesting that additional regulatory elements may be present to sustain *En1* expression.

Here, we used in vivo CRISPR/Cas9 genome editing, gene expression analyses, and enhancer reporter assays to elucidate the regulatory elements responsible for maintaining *En1* expression. We have identified two enhancer elements that cooperate to drive *En1* expression in the E10.5 and E11.5 limb buds that we term late-stage *Engrailed-1*-associated limb-specific enhancers 1 and 2 (*LSEE1* and *LSEE2*). Our data suggest that EN1 binding to *LSEE1&2* provides a mechanism by which *En1* could autoamplify and maintain its expression in mouse limb buds after initial activation by *Maenli*. Strikingly, homozygous deletion of *LSEE1*&*2* together causes morphological limb defects resembling only the postnatal *En1* mutant limb abnormalities (∼98% of mutants) (Loomis et al. 1996; Hanks et al. 1998). In contrast, *Maenli*^−/−^ mice display both the embryonic and postnatal morphological limb defects with complete penetrance, supporting a distinct spatiotemporal contribution of the CREs in controlling *En1* expression. These phenotypic differences are linked, at least in part, to temporal differences in ectopic *Wnt7a* expression in the ventral limb ectoderm. In *En1* and *Maenli* mutants, early ectopic *Wnt7a* expression likely promotes the formation of a secondary ventral AER, resulting in ectopic ventral digits (Loomis et al. 1998; Allou et al. 2021). In contrast, in *LSEE1&2*^−/−^ mutants, *Wnt7a* misexpression seems to occur only at later stages, likely explaining the absence of AER proximoventral expansion and ectopic digit formation. This study uncovers that *En1* is controlled by two transcriptional waves during limb development: an early wave essential for both the AER and dorsal–ventral axis patterning and a late wave instructing dorsal–ventral axis patterning. Importantly, these two transcriptional waves are controlled by *Maenli* and *LSEE1&2*. Our findings highlight how CREs acting in a time-specific manner fine-tune gene function in lineage specification and patterning and contribute to genetic disorders with subtle phenotypic variations.

## Results and Discussion

### Regulatory elements beyond *Maenli* contribute to *En1* expression in E10.5 and E11.5 limb buds

**Figure 1.**
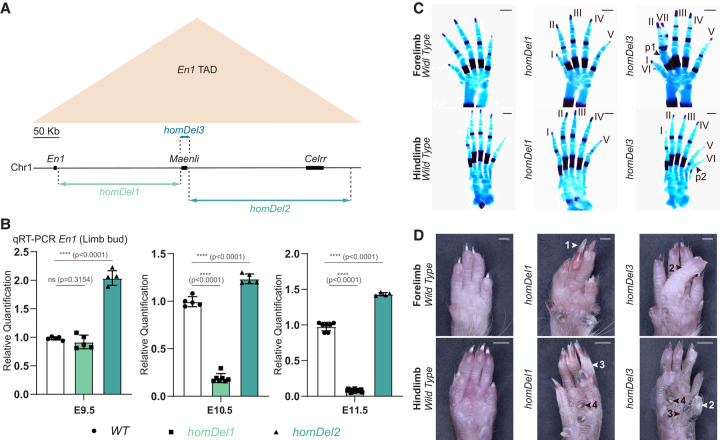
*homDel1* significantly decreases *En1* expression in the limb and causes a double dorsal limb phenotype in mice. (*A*) Schematic representation of the *En1* TAD (chromosome 1: 122,459,752–123,089,561 mm9; beige triangle) and the CRISPR–Cas9 genetic dissection of the *En1* regulatory landscape (*homDel1*, *homDel2*, and *homDel3*). (*B*) Normalized qRT-PCRs of *En1* in E9.5, E10.5, and E11.5 limb embryos show a significant loss of *En1* expression at E10.5 and E11.5 upon deletion of the genomic region between *En1* and *Maenli* (*homDel1*). In contrast, this deletion did not significantly affect *En1* expression at E9.5. Deletion of the genomic region between *Maenli* and the telomeric end of the *En1* TAD (*homDel2*) resulted in a significant increase in *En1* expression at E9.5, E10.5, and E11.5. Data were normalized to wild-type expression; one-tailed *t*-test; data are mean ± SD. *n* = 5 WT, *n* = 5 *homDel1*, *n* = 5 *homDel2* at E9.5; *n* = 5 WT, *n* = 7 *homDel1*, *n* = 5 *homDel2* at E10.5; *n* = 7 WT, *n* = 18 *homDel1*, *n* = 4 *homDel2* at E11.5. (ns) Nonsignificant, (****) *P* < 0.0001. (WT) Wild-type. (*C*) Alcian blue-stained (cartilage) and alizarin red-stained (bone) limbs prepared from wild-type, *homDel1*, and *homDel3* E18.5 embryos. The *homDel1* mutant is indistinguishable from the wild type. The *homDel3* mutant exhibits the presence of a polydactyly (digit VI) and an ectopic ventral digit (VII) fused at the level of phalange 1 in the forelimb and an ectopic ventral digit (VI) fused at the level of phalange 2 in the hind limb. (p) Phalange. Scale bars, 500 µm. *n* = 10 WT, *n* = 8 *homDel1*, *n* = 9 *homDel3*. (*D*) Ventral views of wild-type, *homDel1*, and *homDel3* adult (8 week) forepaws and hind paws illustrating the presence of ectopic ventral nails (1), ectopic ventral digits (2), and ectopic ventral hairs (3). The pigmented metatarsal pads (4) are elongated and hardened, resembling nails. Scale bars: forelimbs, 1000 µm; hind limbs, 2000 µm. *n* = 13 *homDel1*, *n* = 15 *homDel3*.

To investigate whether additional regulatory elements drive *En1* expression in E10.5 and E11.5 limb buds, we performed a genetic dissection of the *En1* regulatory landscape using in vivo CRISPR/Cas9 genome editing. We generated two large homozygous deletions (*homDel1* and *homDel2*) within the *En1* regulatory landscape and a deletion of only the *Maenli* locus (*homDel3*) (Fig. 1A). *homDel1* eliminates the genomic region between *En1* and *Maenli*, while *homDel2* spans from *Maenli* to the telomeric boundary of the *En1* TAD (Fig. 1A; Allou et al. 2021).

In this study, qPCR analyses were performed using *Rps9* as a housekeeping gene to normalize the expression levels of target genes. Additionally, *Gapdh* was used as an endogenous control for all qPCR measurements, and no changes were observed. These analyses showed that *homDel1* and *homDel2* did not affect *Maenli* expression in the E9.5 limb bud (Supplemental Fig. S1A,B). However, while *homDel1* did not significantly affect *En1* expression in the E9.5 limb bud, its expression was significantly decreased at E10.5 and E11.5 (Fig. 1B; Supplemental Fig. S1B,C). In contrast, *homDel2* caused a significant increase in *En1* expression at all three developmental stages (Fig. 1B; Supplemental Fig. S1B,C).

Interestingly, *homDel1* mice showed strong limb malformations resembling those previously described for *En1* mutant (Wurst et al. 1994; Loomis et al. 1996; Hanks et al. 1998; Ma et al. 2024) and *Maenli*^−/−^ (Allou et al. 2021) mice. Notably, these mice exhibited the postnatal (∼4 weeks) morphological limb defects consisting of dorsalization of ventral structures, including ventral or circumferential claws, ventral hairs, loss of pads, hyperpigmentation on the ventral epidermis, and digits flexed dorsally rather than ventrally. However, they did not exhibit the embryonic (<0 days) morphological limb abnormalities, including syndactyly, polydactyly, and ventral digits (Fig. 1C,D; Supplemental Fig. S1D,E). These data suggest that *homDel1* harbors regulatory elements driving *En1* expression in the E10.5 and E11.5 limb buds, with their loss causing only the postnatal *En1* mutant limb phenotype.

### A second lncRNA locus, *Lmer*, seems to be dispensable for limb-specific *En1* expression

**Figure 2.**
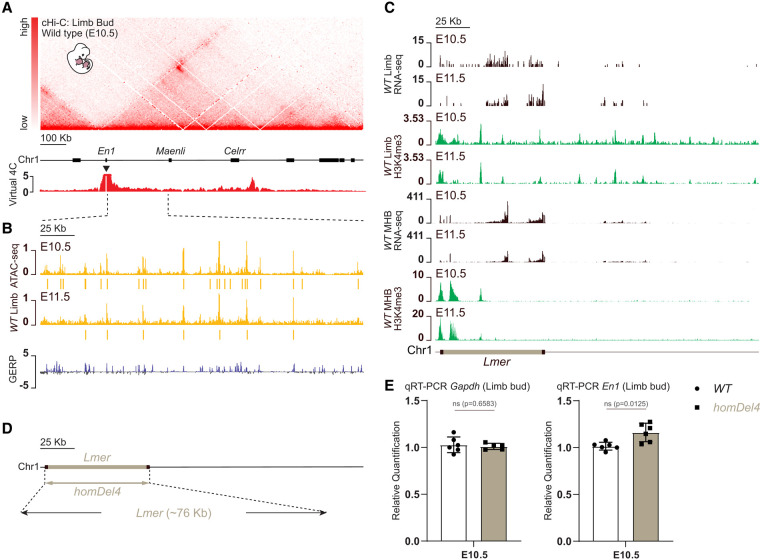
*Lmer* is likely dispensable for *En1* expression regulation during limb development. (*A*) A cHi-C map of the mouse limb buds at E10.5 shows a large TAD encompassing the *En1* gene and the *Maenli* and *Celrr* lncRNA loci (chromosome 1: 122,459,752–123,089,561 mm9). A virtual 4C profile using the *En1* promoter as the viewpoint (black triangle) shows no prominent interaction between *En1* and the genomic region upstream of *Maenli*. *n* = 2 biologically independent wild-type replicates with two technical replicates each. (*B*) ATAC reads from wild-type (WT) E10.5 and E11.5 mouse limb buds are shown. Peak calling identifying genomic regions that are enriched in aligned ATAC reads (to a statistically significant extent) compared with background levels is also shown. *n* = 2 biologically independent wild-type replicates. Vertebrate conservation is measured using the genomic evolutionary rate profiling (GERP) method. (*C*) Poly(A)^+^ RNA-seq and H3K4me3 ChIP-seq profiles of E10.5 and E11.5 mouse limb buds and the midbrain/hindbrain junction show the presence of the lncRNA locus *Lmer*. *n* = 2 biologically independent wild-type replicates. (*D*) Schematic representation of the CRISPR–Cas9 genetic deletion involving the lncRNA locus *Lmer* (*homDel4*). (*E*) Normalized qRT-PCRs of *Gapdh* and *En1* in E10.5 mouse limb embryos show no significant changes in *Gapdh* and *En1* expression upon deleting the lncRNA locus *Lmer* (*homDel4*). Data were normalized to wild-type expression; one-tailed *t*-test; data are mean ± SD. *n* = 6 WT, *n* = 6 *homDel4* for *En1* expression quantification; *n* = 6 WT, *n* = 5 *homDel4* for *Gapdh* expression quantification. (ns) Nonsignificant. (WT) Wild type.

To identify potential regulatory elements within *homDel1* driving *En1* expression at E10.5 and E11.5, we first performed capture Hi-C (cHi-C) in E10.5 limb buds (Fig. 2A). We examined chromatin interactions between *homDel1* and *En1* using the *En1* promoter as the viewpoint by deriving a virtual 4C profile from the cHi-C data. However, despite strong interactions between the *En1* promoter and the telomeric end of the *En1* TAD, we found no prominent specific interaction with a particular region in *homDel1* (Fig. 2A).

We then searched for putative enhancers and transcripts in *homDel1* by analyzing previously published (Paliou et al. 2019; Allou et al. 2021) assays for transposase-accessible chromatin followed by sequencing (ATAC-seq) data in E10.5 and E11.5 limb buds. Interestingly, the *homDel1* region contained seven to 23 putative enhancers defined by ATAC-seq peaks (Fig. 2B). Additionally, we performed poly(A)^+^ RNA sequencing (RNA-seq) in wild-type E10.5 and E11.5 limb buds, somites, and midbrain/hindbrain junctions. These analyses revealed the presence of a transcript in the limb buds and the midbrain/hindbrain junction within *homDel1* that was not detected in the somites (Fig. 2C; Supplemental Fig. S2A). PhyloCSF analysis showed that this transcript lacks protein-coding potential, suggesting that it is a lncRNA (Supplemental Fig. S2B). This is further supported by its low nucleotide sequence conservation, with only 28% similarity between mice and humans, and overall low conservation across other species (Supplemental Fig. S2B). We named this lncRNA locus *Lmer* (limb midbrain/hindbrain junction-expressed RNA) (Fig. 2C).

The mouse *Lmer* locus encompasses 11 exons spanning ∼76 kb and is located ∼6 kb downstream from the 3′ end of *En1* (Fig. 2D). Its transcriptional start site (TSS) overlaps with H3K4me3 marks in E10.5 and E11.5 limb buds and midbrain/hindbrain junctions (Fig. 2C). We tested whether the *Lmer* locus is required for *En1* expression and limb development in vivo by deleting it (*homDel4*) (Fig. 2D). This deletion did not seem to significantly affect *En1* expression in E10.5 limb buds (Fig. 2E). Thus, the mouse *Lmer* locus appears to be dispensable for the correct timing and location of *En1* expression during limb development.

### Subtle differences in limb defects reveal a critical region for regulating late-stage *En1* expression

We next hypothesized that the genomic region between *Lmer* and *Maenli* (∼157 kb) contains regulatory elements controlling *En1* expression in E10.5 and E11.5 limb buds. This genomic region contains four to 13 putative enhancers in the limbs defined by ATAC-seq peaks (Fig. 2B). To determine the position of potential *En1* regulatory elements within this large genomic region, we analyzed the phenotypic differences between three mutants (*homDel1* and *homDel3*, generated in this study, and *homDel27*, generated in our previous study [Allou et al. 2021]) (Supplemental Fig. S3A). Interestingly, while all of these mutants exhibited limb abnormalities resembling those of *En1* mutant mice (complete penetrance) (Wurst et al. 1994; Loomis et al. 1996; Hanks et al. 1998; Ma et al. 2024), they showed small but noticeable phenotypic differences.

*homDel27* mice (with a deletion of the *Maenli* locus and ∼9 kb of the adjacent noncoding DNA sequences) exhibited syndactyly with or without polydactyly (embryonic limb defects) in all forelimbs and ectopic ventral or circumferential nails (postnatal limb abnormalities) in all forelimb and hind limb digits (*n* = 16) (Allou et al. 2021). In *homDel3* mutants, however, the syndactyly with or without polydactyly was observed in approximately one forelimb of each embryo, and ectopic ventral or circumferential nails were observed in approximately two to three digits of each limb (*n* = 15). Finally, in *homDel1* mice, the syndactyly and polydactyly were absent, with ectopic ventral or circumferential nails present in all forelimb and hind limb digits (*n* = 13) (Supplemental Fig. S3A). These observations suggest temporal differences in *En1* expression between the three mutants during limb development.

We tested this by quantifying *En1* expression at E9.5, E10.5, and E11.5 in *homDel3* and *homDel27* limb buds using RNA-seq and *homDel3* limb mutants using qRT-PCR (Supplemental Fig. S3B,C). *homDel3* embryonic limbs showed an almost complete loss of *En1* expression at E9.5 and reduced levels at E10.5 and E11.5 (∼35% and ∼62% of wild type levels, respectively) (Supplemental Fig. S3B,C). Moreover, RNA-seq in *homDel27* limb buds showed a complete loss of *En1* expression at all three developmental stages (Supplemental Fig. S3C). These data demonstrate that *homDel27* contains the complete regulatory information required to control *En1* expression during limb development. In contrast, deletion of *Maenli* alone (*homDel3*) leads to only a partial loss of *En1* expression in E10.5 and E11.5 limb buds, confirming that additional regulatory elements maintain *En1* expression. In combination with the results from the *homDel1* mutants (Fig. 1B; Supplemental Fig. S1B,C), these findings suggest that putative regulatory elements located within the overlapping genomic region between *homDel1* and *homDel27* control *En1* expression in the limb buds after E9.5.

### Late-stage enhancers are required for proper dorsal–ventral limb patterning

**Figure 3.**
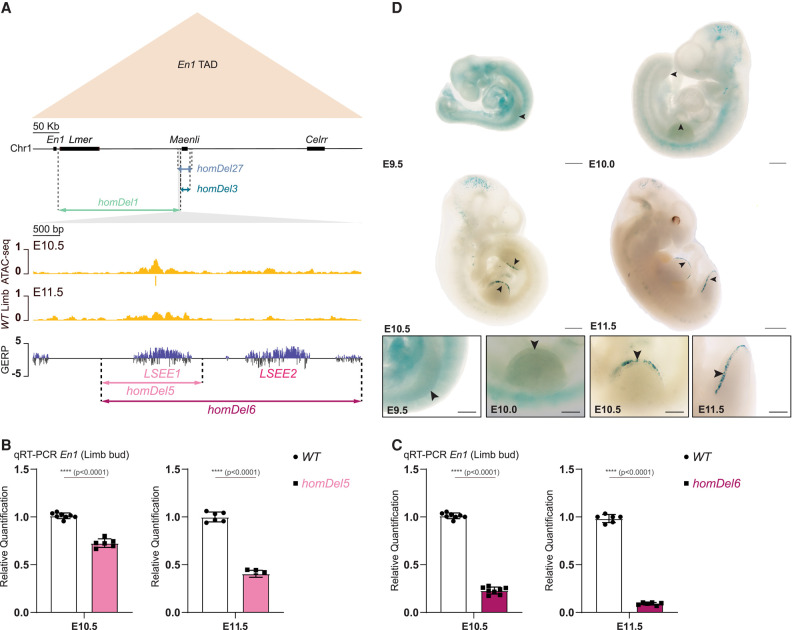
*LSEE1&2* enhancers cooperate to drive *En1* expression in E10.5 and E11.5 mouse limb buds. (*A*) Schematic representation of the *En1* TAD (chromosome 1: 122,459,752–123,089,561 mm9; beige triangle) and the CRISPR–Cas9 genetic deletions (*homDel1*, *homDel3*, and *homDel27*) affecting *En1* limb expression and causing abnormal limb bud dorsoventral patterning in mice. The overlapping genomic region between *homDel1* and *homDel27* is highlighted in gray, and an enlarged version of the region is shown. ATAC reads from wild-type (WT) E10.5 and E11.5 mouse limb buds indicate the presence of a putative enhancer element within the overlapping genomic regions. Vertebrate conservation is measured using the genomic evolutionary rate profiling (GERP) method, which indicates the presence of two highly conserved noncoding DNA elements within the overlapping genomic regions. Finally, a schematic representation of the CRISPR–Cas9 genetic dissection of the overlapping genomic regions (*homDel5* and *homDel6*) is shown. (*B*) Normalized qRT-PCRs of *En1* in E10.5 and E11.5 mouse limb embryos show a significant loss of *En1* expression upon deleting the single putative enhancer *LSEE1* (*homDel5*). Data were normalized to wild-type expression; one-tailed *t*-test; data are mean ± SD. *n* = 8 WT, *n* = 6 *homDel5* at E10.5; *n* = 6 WT, *n* = 6 *homDel5* at E11.5. (ns) Nonsignificant, (****) *P* < 0.0001. (WT) Wild-type. (*C*) Normalized qRT-PCRs of *En1* in E10.5 and E11.5 mouse limb buds isolated from *homDel6* embryos show a significant loss of *En1* expression at both developmental stages, with an almost complete loss at E11.5. Data were normalized to wild-type expression; one-tailed *t*-test; data are mean ± SD. *n* = 8 WT, *n* = 8 *homDel6* at E10.5; *n* = 6 WT, *n* = 6 *homDel6* at E11.5. (ns) Nonsignificant, (****) *P* < 0.0001. (WT) Wild-type. (*D*) *LSEE1&2* regulatory elements drive limb-specific *LacZ* expression in E10.5 and E11.5 mouse limb buds but not in E9.5 and E10.0 mouse limb embryos; they exhibit temporally restricted enhancer activities in the developing limb bud. Scale bars: whole embryos at E11.5, 1000 µm; whole embryos at E9.5, E10.0, and E10.5, 500 µm; high-magnification views of limbs, 200 µm. *n* = 17 embryos at or before E10.0; *n* = 4 embryos at or after E10.5.

The overlapping ∼6 kb genomic region between *homDel1* and *homDel27* contains one putative enhancer element defined by ATAC-seq (Fig. 3A). An ∼2 kb deletion of this putative enhancer (*homDel5*) led to a significant decrease in *En1* expression—by ∼23% and ∼60% in E10.5 and E11.5 mutant limb buds, respectively (Fig. 3A,B; Supplemental Fig. S4A). This reduction was less severe than the one observed in E10.5 and E11.5 *homDel1* limb mutants (Fig. 1B; Supplemental Fig. S1B,C). Therefore, we next generated an ∼4 kb deletion covering both conserved noncoding DNA elements (*homDel6*) as defined by vertebrate conservation measured using genomic evolutionary rate profiling (GERP) (Fig. 3A). This led to a significant reduction in *En1* expression in E10.5 and E11.5 limb buds (∼77% and ∼91% decrease, respectively) (Fig. 3C; Supplemental Fig. S4B). *homDel6* mutants displayed the same reduction in *En1* expression in E10.5 and E11.5 limb buds as *homDel1* mutants (Figs. 1B, 3C; Supplemental Figs. S1B,C, S4B). These data indicate that the deleted region (*homDel6)* contains two CREs that cooperate to drive *En1* expression in the E10.5 and E11.5 limb buds. We examined the epigenetic signature of these CREs by analyzing previously published (Allou et al. 2021) and newly generated H3K4me1, H3K4me3, and H3K27ac chromatin immunoprecipitation followed by sequencing (ChIP-seq) data in E9.5, E10.5, and E11.5 limb buds (Supplemental Fig. S4C). Unlike the *Maenli* TSS, which is marked by H3K4me1, H3K4me3, and H3K27ac active marks at E9.5, the identified CREs overlap with H3K4me1 but lack the H3K27ac mark associated with active enhancers at E10.5. At E11.5, one of the CREs displays a weak accumulation of H3K27ac and H3K4me3 active marks, suggesting that the identified CREs may act as enhancers (Supplemental Fig. S4C). We thus tested the enhancer activity of these regulatory elements from E9.5 to E11.5 using an in vivo enhancer reporter assay (Supplemental Fig. S4D–F). The CREs showed strong enhancer expression in the AER of the limb bud at E10.5 and E11.5 (*n* = 4) (Fig. 3D). However, no enhancer activity was detected in E9.5 and E10.0 limb embryos (*n* = 17) (Fig. 3D; Supplemental Fig. S4G), indicating that these CREs act as *En1* enhancers in the limb specifically at E10.5 and E11.5. We named these enhancer elements *LSEE1* and *LSEE2* (late-stage *Engrailed-1*-associated limb-specific enhancers 1 and 2), respectively (Fig. 3A).

**Figure 4.**
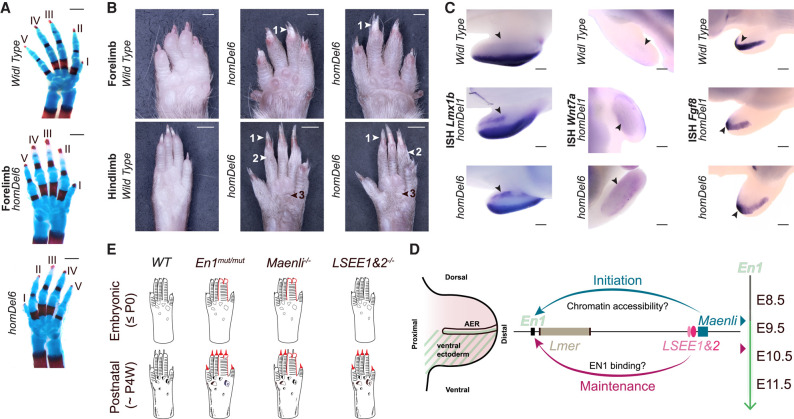
*LSEE1&2* are required for proper dorsal–ventral limb patterning. (*A*) Alcian blue-stained (cartilage) and alizarin red-stained (bone) limbs prepared from wild-type and *homDel6* E17.5 embryos. The *homDel6* mutants are indistinguishable from the wild type. Scale bars, 500 µm. *n* = 4 WT, *n* = 8 *homDel6*. (*B*) Ventral views of wild-type and *homDel6* adult (6 week) forepaws and hindpaws illustrating the presence of ectopic ventral nails (1) and ectopic ventral hairs (2). The pigmented metatarsal pads (3) are elongated and hardened, resembling nails. Scale bars: forelimbs, 1000 µm; hind limbs, 2000 µm. *n* = 7 *homDel6*. (*C*) Whole-mount in situ hybridization (WISH) of E10.5–E12.5 wild-type, *homDel1*, and hom*Del6* embryos probed with *Fgf8*, *Lmx1b*, or *Wnt7a*. Limb mutants display ectopic ventral expression of the dorsalizing genes *Lmx1b* and *Wnt7a* (indicated by black arrowheads). However, they do not display the aberrant proximoventral expansion of the *Fgf8* expression domain in the AER (indicated by black arrowheads), as reported previously for *Maenli* homozygous inactivation and *homDel27* (Allou et al. 2021). Scale bars, 200 µm. *n* ≥ 3. E12.5 WT, E11.5 *homDel1*, and E11.5 *homDel6 Lmx1b* embryos; E11.5 WT, E11.5 *homDel1*, and E11.5 *homDel6 Wnt7a* embryos; and E10.5 WT, E11.5 *homDel1*, and E11.5 *homDel6 Fgf8* embryos are shown. (*D*) During limb development, *Maenli*, *LSEE1*, and *LSEE2* fine-tune the temporal expression of *En1*. *Maenli* licenses *En1* expression specifically at E9.5, likely by rewiring chromatin accessibility. *LSEE1*&*2* cooperate to drive *En1* expression specifically at E10.5 and E11.5, likely through EN1 binding. *Maenli* is required for both dorsal–ventral patterning and AER formation and maturation, whereas *LSEE1*&*2* are critical only for proper dorsal–ventral patterning. (*E*) Schematic representation of *Maenli*^−/−^- and *LSEE1&2*^−/−^-associated limb phenotypes. *Maenli*^−/−^ and *LSEE1&2*^−/−^ mice exhibit limb defects recalling those of *En1* mutant mice. However, while *Maenli*^−/−^ mutants exhibit the embryonic and postnatal morphological limb defects, *LSEE1&2*^−/−^ mutants exhibit only the postnatal morphological limb abnormalities (∼98% of mutants). Differential phenotypic features between *En1* mutant, *Maenli*^−/−^, and *LSEE1&2*^−/−^ mice include the syndactyly with or without polydactyly and ectopic ventral nails (highlighted in red). (P0) Postnatal day 0, (P4W) 4 weeks postnatally.

Similar to *homDel1* mutants, seven out of seven *homDel6* mutants displayed only the postnatal limb malformations (Fig. 4A,B; Supplemental Fig. S5A). A single *homDel6* mutant (*n* = 1 out of 15) displayed a syndactyly in one of the forelimbs (Fig. 4A,B; Supplemental Fig. S5A), indicating a very low penetrance of the embryonic phenotype in the mutants lacking *LSEE1&2* (*homDel1* and *homDel6*; *n* = 1 out of 36). The loss of *En1* in E11.5 *homDel1* and *homDel6* limb mutants resulted in an ectopic ventral expression of the dorsal marker genes *Lmx1b* and *Wnt7a*, as described previously for *En1* mutants, *homDel27*, and *Maenli* homozygous inactivation mice (Fig. 4C; Loomis et al. 1998; Allou et al. 2021). Nevertheless, this loss did not result in a proximoventral expansion of the AER marker gene *Fgf8*, unlike what was reported previously for *En1* mutants, *homDel27*, and *Maenli* homozygous inactivation mice (Fig. 4C; Loomis et al. 1998; Allou et al. 2021). This absence of secondary ventral apical ectodermal ridges in embryos lacking *LSEE1&2* could be explained by a temporal effect on ectopic *Wnt7a* expression in the ventral limb ectoderm of these mutants. Indeed, RNA-seq data show that *Wnt7a* expression seems to be gained already at E9.5 in *homDel3* (*Maenli*^−/−^) and *homDel27* (*Maenli*^−/−^*;LSEE1&2*^−/−^) limb mutants but only gained at E11.5 in *homDel1* limb mutants (*LSEE1&2*^−/−^) (Supplemental Fig. S5B). *Lmx1b* expression seems to be gained at E11.5 in all limb mutants (Supplemental Fig. S5C). Altogether, these data indicate that *LSEE1&2* are critical for directing the development of ventral limb structures.

We next examined two main hypotheses investigating the interplay between *Maenli*, *LSEE1&2*, and EN1 in regulating *En1* expression during limb development. First, given that *LSEE1&2* are positioned ∼2.5 kb upstream of *Maenli*, we tested whether the initial transcriptional activity of *Maenli* at E9.5 is needed for the downstream activity of *LSEE1&2*. We generated an ∼235 kb homozygous inversion (*homInv*), relocating the enhancers from the 3′ end of *Maenli* to the 3′ end of *En1* (∼4 kb downstream) (Supplemental Fig. S5D). This inversion had no significant impact on *En1* expression in E11.5 limb buds (Supplemental Fig. S5E), suggesting that *LSEE1&2* enhancer activity may not depend on the initial transcriptional elongation through the *Maenli* locus. Second, we hypothesized that residual *En1* expression (∼8%) in the absence of *Maenli* alone (*homDel3*) could be sufficient to facilitate a low level of *LSEE1&2* enhancer activity by EN1 binding to its enhancers, leading to the partial recovery of *En1* expression at later stages (Supplemental Fig. S3B,C). We searched for EN1 transcription factor binding sites within *LSEE1&2* using the reported JASPAR binding motif (Rauluseviciute et al. 2024) and identified four conserved potential EN1 binding sites within *LSEE1&2* (Supplemental Fig. S5F). Interestingly, two of the conserved EN1 binding motifs were located in the genomic region that is enriched in aligned ATAC reads within *LSEE1* (Supplemental Fig. S5F). These data suggest that *En1* expression might be maintained during limb development through a positive feedback loop where EN1 binds to its own enhancers.

### Summary and perspectives

Developmental genes are often regulated by multiple enhancers exhibiting similar spatiotemporal outputs (Dunipace et al. 2019). Here, we show that *En1* in the limb is regulated by an early-acting CRE, the enhancer-associated lncRNA locus *Maenli*, initiating its expression and late-stage-specific enhancers *LSEE1&2*, which maintain its expression. Hence, *En1* is regulated by multiple CREs displaying distinct spatiotemporal outputs. Individual loss of *Maenli* and *LSEE1&2* causes a temporal loss of *En1* expression in the limb, which alters the spatiotemporal expression of key genes governing axial patterning pathways, resulting in distinct phenotypes.

Deleting the genomic region between *Maenli* and the telomeric end of the *En1* TAD (*homDel2*) significantly increased *En1* expression during limb development (Fig. 1B; Supplemental Fig. S1B,C). This deletion shifts the telomeric TAD boundary closer to *En1* CREs, possibly increasing their contact frequency with the *En1* promoter, leading to its upregulation. In contrast, removal of the region between *En1* and *Maenli* (*homDel1*) significantly reduced *En1* expression in the E10.5 and E11.5 limb buds (Fig. 1B; Supplemental Fig. S1B,C). In this region, we found another lncRNA locus, *Lmer*, which we thought may regulate *En1* at later stages (Fig. 2C). However, we found that *Lmer* appears to be dispensable for limb development (*homDel4*) (Fig. 2D,E). Instead, *Lmer* is highly expressed in the midbrain/hindbrain junction, where *En1* is also expressed and is essential for its development (Fig. 2C; Ma et al. 2024). How *En1* expression is controlled in this linear structure between the two brain regions is unknown. Hence, it is tempting to speculate that *Lmer* controls *En1* expression in the midbrain/hindbrain junction.

With further deletions and enhancer reporter assays, we identified an ∼4 kb genomic region containing the *LSEE1&2* enhancers required for maintaining *En1* expression after E9.5 (Fig. 3A–D; Supplemental Figs. S3A–C, S4A–G). Homozygous deletion of *LSEE1&2* resulted in an up to 91% loss of *En1* expression in E11.5 limb buds (Fig. 3C). Although we do not know whether *LSEE1&2* cooperate redundantly, additively, or synergistically, it seems that their function may depend on initial activation by *Maenli*. Indeed, deletion of *Maenli* and *LSEE1&2* together (*homDel27*) fully ablates *En1* expression at all limb developmental stages investigated here (Supplemental Fig. S3C). However, the presence of only *LSEE1&2* in *homDel3* (homozygous deletion of *Maenli*) causes an ∼92% reduction in *En1* expression at E9.5, but it recovers to only ∼62% by E11.5 (Supplemental Fig. S3B,C). Moreover, the enhancer reporter assays and histone modification ChIP-seq data show that *LSEE1&2* are first activated at E10.5 after the initial activation of *Maenli* at E9.5 (Fig. 3D; Supplemental Fig. S4C–G). This suggests a temporal interplay of CRE activity at the *En1* locus where the activity of *Maenli* is required for later enhancer activity. Such a complex regulatory architecture with interdependencies on multiple CREs in vivo has been reported for only a few genes including *Sox9* (Gonen et al. 2018). *Sox9* plays a crucial role in sex determination, where its transcription in the gonads relies on an enhancer cascade involving the activity of an early enhancer that is required for later enhancer activation (Gonen et al. 2018). A similar cascade may sustain *En1* expression in the limb through a positive feedback loop where, after initial activation by *Maenli*, EN1 binds to its own enhancers, *LSEE1&2* (Supplemental Fig. S5F). Such a positive feedback mechanism has been reported for LMX1B, where it binds to two conserved *Lmx1b*-associated CREs that in turn amplify *Lmx1b* expression during limb dorsalization (Haro et al. 2021).

*En1* mutation experiments from the 1990s revealed distinct embryonic and postnatal limb phenotypes in mice (Wurst et al. 1994; Loomis et al. 1996; Hanks et al. 1998; Ma et al. 2024). Our previously reported mutant mice that lack *Maenli* and the here identified enhancers *LSEE1&2* (*homDel27*) displayed syndactyly with or without polydactyly (embryonic limb defects) in all forelimbs and ectopic ventral nails (postnatal limb abnormalities) in all forelimb and hind limb digits (Allou et al. 2021). However, *homDel3* mice carrying a deletion of solely *Maenli* exhibit a syndactyly with or without polydactyly in approximately one forelimb of each embryo and ectopic ventral nails in approximately two to three digits of each limb (Fig. 1C,D; Supplemental Fig. S1D,E). This suggests that *LSEE1&2* can partially rescue *En1* expression in the absence of *Maenli*. Interestingly, ∼98% of the mutant mice carrying homozygous deletions that include *LSEE1&2* (*homDel1* and *homDel6*) display only the postnatal morphological limb phenotype (Figs. 1C,D, 4A,B; Supplemental Figs. S1D,E, S5A), indicating spatiotemporal differences in the regulatory genes governing the axial patterning pathways in the limb (Loomis et al. 1998; Delgado and Torres 2017). While all mutants exhibited ectopic *Wnt7a* expression in the ventral limb ectoderm (Fig. 4C; Allou et al. 2021), they seem to gain *Wnt7a* at different time points during limb development, correlating with phenotypic variation. In *homDel3* and *homDel27* mice, *Wnt7a* seems to be gained at or after E9.5, while in *homDel1* mice, *Wnt7a* seems to be gained at E11.5 (Supplemental Fig. S5B). Moreover, *Fgf8*, a key gene of the proximal–distal axis, did not display a proximoventral expansion of the AER in *homDel1* and *homDel6* (Fig. 4C), unlike what was reported previously for *Maenli* homozygous inactivation and *homDel27* (Allou et al. 2021). *homDel1* and *homDel6* mutants do not exhibit embryonic limb abnormalities—either ectopic ventral digits (*n* = 36 out of 36) or syndactyly/polydactyly (*n* = 35 out of 36). A single case of syndactyly observed in an *LSEE1&2*^−/−^ mutant may be due to an AER defect or interdigit mesenchyme cell death issues at E12.0 (Lorda-Diez et al. 2015) or may be a “false positive phenotype” due to the influence of tetraploid complementation (George et al. 2007).

Together, these data support a model in which temporal regulation of CREs underlies phenotypic differences between *Maenli*^−/−^, *LSEE1&2*^−/−^, and *Maenli*^−/−^*;LSEE1&2*^−/−^ mutants. Specifically, *Maenli* and *LSEE1&2* coordinate two distinct transcriptional waves that regulate *En1* expression during limb development. The early wave is essential for the AER and dorsal–ventral axis patterning, as displayed by the proximoventral expansion of *Fgf8* and ectopic *Wnt7a* and *Lmx1b* expression in the ventral limb ectoderm of *Maenli* mutants (Allou et al. 2021). The late wave is essential for dorsal–ventral axis patterning, as shown by the ectopic *Wnt7a* and *Lmx1b* expression in the ventral limb ectoderm of *LSEE1&2*^−/−^ mutants, which do not show either proximoventral expansion of *Fgf8* (Fig. 4C) or skeletal digit abnormalities in ∼98% of mice (Figs. 1C,D, 4A,B; Supplemental Figs. S1D,E, S5A). Nevertheless, the presence of syndactyly in a single *LSEE1&2*^−/−^ mutant indicates that *En1* may play a role in correct AER formation when expressed at E10.5–E11.5. However, other compensatory mechanisms are present, such as the one suggested for EN2 biochemical function (Hanks et al. 1995), explaining the nonessential role of *En1* at these developmental stages and the absence of skeletal digit abnormalities in ∼98% of *LSEE1&2*^−/−^ mutants. Such a spatiotemporal role of enhancers in developmental patterning has been reported for *Hox* genes, which display a unique spatial and temporal regulation during development (Hubert and Wellik 2023). The *HoxA* and *HoxD* clusters are regulated by enhancers positioned in their flanking domains that work to coordinate sequential gene activation during limb development. In a first wave, enhancers in the 3′ domain activate *Hox* genes, patterning the stylopod and zeugopod. Then, in a second wave, enhancers in the 5′ domain activate *Hox* genes to pattern the digits (Hubert and Wellik 2023).

Our study opens several questions that can be addressed in the future. First, it remains unclear whether additional regulatory elements beyond *Maenli* and *LSEE1*&2 contribute to the precise control of *En1* during limb development. Although *Maenli* and *LSEE1&2* constitute the primary drivers of early and late *En1* expression, potential cooperativity with other regulatory elements within the *En1* TAD cannot be ruled out. Such cooperative interactions may manifest as changes in spatial expression patterns rather than in transcript abundance and could be assessed in embryos carrying deletions of yet uncharacterized CREs. Second, whether the *Lmer* locus fine-tunes *En1* transcript levels in the limb bud could be further studied. Our single-time-point qPCR analysis shows a modest upregulation at E10.5 (Fig. 2E). While not statistically significant, these data suggest that additional experiments are needed to determine whether *Lmer* contributes to *En1* transcriptional regulation in the limb. Moreover, although this study focuses entirely on limb development, it would be of interest to investigate *Lmer*’s function in the midbrain/hindbrain boundary. Third, we demonstrated that *LSEE1&2* function as enhancers in the developing limb bud, but it is still unclear whether they operate without the canonical chromatin mark H3K27ac or whether the mark is simply undetected. A recent study by Mannion et al. (2025) compared functionally validated in vivo enhancers with matched chromatin profiling data and showed that many validated enhancers lack canonical chromatin marks. Our data for *LSEE1&2* are consistent with these findings and further indicate that open chromatin may serve as a more reliable starting point for identifying potential enhancer activity. Assays such as ChIP-seq may fail to detect chromatin marks of enhancers with narrow temporal dynamics, low activity, or activity restricted to small cell populations. Therefore, reassessing H3K27ac enrichment at *LSEE1&2* using fluorescence activated cell sorting (FACS)-sorted EN1-positive ectodermal cells would be informative. Finally, single-cell profiling approaches will be beneficial for defining the cooperativity and precise molecular mechanisms by which *Maenli*, *LSEE1&2*, and other potential CREs drive *En1* expression during embryogenesis (Fig. 4D).

In summary, this study provides new insights into *En1* gene regulation and function during limb development. *En1* has two critical spatiotemporal roles. At early stages, it is required for proper dorsal–ventral patterning as well as AER formation and maturation under the control of *Maenli* (Supplemental Fig. S5G). At later stages, its function is essential for proper dorsal–ventral patterning, which is associated with *LSEE1&2* enhancers (Supplemental Fig. S5G). Loss of the early (*Maenli*) versus late (*LSEE1&2*) CREs results in distinct phenotypes (Fig. 4E; Supplemental Fig. S5G,H), extending previous reports showing distinct phenotypic effects of time-specific CRE deletions (Gonen et al. 2018; Dunipace et al. 2019; Kvon et al. 2021; Hubert and Wellik 2023; Rouco et al. 2026). Our findings have important implications for congenital limb malformations, which are common (Giele et al. 2001) and frequently isolated. The diagnostic yield in these cases remains low due to, at least in part, a focus on studying coding sequences. Our previous findings and others have shown that tissue-specific CREs are potential candidates to explain these isolated limb malformations (Lettice et al. 2002; Dathe et al. 2009; Birnbaum et al. 2012; Anderson et al. 2016; Isoda et al. 2017; Skuplik et al. 2018; Ritter et al. 2019; Allou et al. 2021; Haro et al. 2021). The findings of this study underscore the significance of time-specific CREs in fine-tuning gene function in developmental patterning and lineage commitment, explaining subtle differences in complex disease phenotypes.

## Materials and methods

### Generation of CRISPR/Cas9 genetic deletions and inversion

Genome-editing experiments were performed as described previously (Kraft et al. 2015). sgRNAs were designed within close proximity of the deletion or inversion breakpoints using the Benchling platform (https://www.benchling.com) to obtain candidate sgRNA sequences. To minimize off-target effects, guide sequences were chosen to have a quality score >95%. Complementary strands were annealed, phosphorylated, and cloned into the BbsI site of the pX459 CRISPR/Cas vector (Addgene). The sequences of all sgRNAs used in this study are listed in Supplemental Table S1. Four-hundred-thousand G4 embryonic stem cells (ESCs; 129/Sv × C57BL/6 F1 hybrid) (George et al. 2007) were seeded on CD1 mouse embryonic fibroblast (MEF) feeders and cultured under standard ESC culture conditions. The cells were transfected with 8 µg of each pX459-sgRNA construct using FuGENE HD reagent (Promega) under the manufacturer's conditions. After 12 h, cells were split, transferred into DR4 puromycin-resistant feeders, and selected with puromycin at a final concentration of 2 µg/mL for 48 h. Clones were then grown for an additional 5–6 days, picked, and transferred onto 96 well plates on CD1 feeders. After 48 h of culture, plates were split into triplicates—two for freezing and one for growth and DNA harvesting. All clones were genotyped by PCR and quantitative PCR (qPCR) analyses. Positive clones were thawed and grown on CD1 feeders until they reached an average of 4 million cells. Three vials were frozen, and DNA was harvested from the rest of the cells to confirm the genotyping results. The sequencing breakpoints of all the homozygous deletions and inversions generated in this study are listed in Supplemental Table S2.

ESCs and feeder cells were tested for *Mycoplasma* contamination using the MycoAlert detection kit (Lonza) and MycoAlert assay control set (Lonza). DR4 and CD1 feeder cell lines were directly produced from mouse embryos originating from DR4 and CD1 mice mattings, respectively.

### Genotyping by polymerase chain reaction and sequencing

Primers were designed at a distance of 400–500 bp from each cutting site on both sides of sgRNA targets. Each allele thus has a set of four primers: F1(fwd)/R1(rev) amplifying one targeted site and F2(fwd)/R2(rev) amplifying the other targeted site. Deletions, duplications, and inversions were detected using the following set of primers: F1/R2, F2/R1, and F1/F2 and R1/R2, respectively. We genotyped each clone by running the PCR products on agarose gels and comparing PCR amplicon sizes. Clones showing evidence of the presence of the desired genomic rearrangement were further genotyped by Sanger sequencing. The sequences of all genotyping primers used in this study are listed in Supplemental Table S1.

### Genotyping by quantitative PCR

qPCR was performed on genomic DNA using the QuantStudio 7 Flex real-time PCR system (v1.7.1; Applied Biosystems). For the genotyping of copy number variations (CNVs), we designed different sets of primers. qPCR was carried out in a total volume of 20 µL containing 10 µL of Power SYBR Green master mix (Applied Biosystems), 0.4 µM concentration of each primer, and 10 ng of genomic DNA. Thermal cycling conditions were for 20 sec at 95°C, followed by 40 cycles of 3 sec at 95°C and 30 sec at 60°C. A noncoding region was selected as the control amplicon. Validation experiments demonstrated that amplification efficiency of the control and all target amplicons was approximately equal. All samples were run in triplicate. The dosage of each amplicon relative to the control amplicon and normalized to wild-type DNA was determined using the 2^−ΔΔ*Ct*^ method.

### Transgenic mouse strains

Mice were generated from the corresponding ESC clone by diploid or tetraploid aggregation (Artus and Hadjantonakis 2011) after thawing a frozen ESC vial seeded on CD1 feeders and growing the embryos for 2 days. Female mice of the CD1 strain were used as foster mothers.

All animal procedures were conducted as approved by the local authorities (Landesamt für Gesundheit und Soziales Berlin) under license numbers G0243/18 and G0176/19. Routine bedding, food, and water changes were performed. Mice were housed in a centrally controlled environment with a 12 h light/12 h dark cycle at 20°C–22.2°C and 30%–50% humidity. All animal experiments followed all relevant guidelines and regulations.

### Enhancer reporter assay

The *LSEE1&2* sequence was amplified by PCR. The SYN72 vector (pCMV-SynA [Addgene plasmid 174860]) was linearized by digestion with *PacI* (NEB), and the *LSEE1&2* insert was integrated using the Gibson Assembly cloning kit (NEB). The assembled construct was confirmed by Sanger sequencing. The full sequence of the plasmid is provided as a FASTA file (Supplemental Fasta Files S1, S2). For genomic integration, 2 µg of ΦC plasmid (pCMVInt [Addgene plasmid 18935]) and 4 µg of the SYN72-*LSEE1&2* construct were coelectroporated into 0.5 × 10^6^ mouse embryonic stem cells using the Neon NxT electroporation system (Thermo Fisher Scientific) with the following settings: 1100 V, 1 msec pulse width, and a single pulse. The primers used for genotyping of the ESC clones are listed in Supplemental Table S1.

Transgenic embryos were collected at E9.5, E10.5, and E11.5 and fixed for 20 min in 4% paraformaldehyde/PBS. Embryos were incubated in X-gal solution (PBS containing 2 mM MgCl_2_, 0.01% sodium deoxycholate, 0.02% nonidet P-40 supplemented with 0.5 mg/mL X-gal, 5 mM potassium ferrocyanide, 5 mM potassium ferricyanide) for 5 h at 37°C. Afterward, embryos were washed with PBS and refixed in 4% PFA for 20 min. Images were acquired using a Zeiss SteREO Discovery.V12 with a cold light source CL9000 microscope and a Leica DFC420 digital camera.

### Data availability

All data sets have been deposited in the Gene Expression Omnibus (GEO) database and are accessible under accession numbers GSE277393, GSE277394, and GSE277395 (or were published previously under accession nos. GSE137335, GSE84795, and GSE82568).

## Supplemental Material

Supplement 1

Supplement 2

Supplement 3

Supplement 4

Supplement 5

Supplement 6
