# Supplementary material for: Temporal loss of En1 during limb development causes distinct phenotypes
Source: Genes Dev. 2026 May 1;40(9-10):627–37. doi: 10.1101/gad.353542.125 (PMC13138323; doi:10.1101/gad.353542.125)
Supplement: Supplement 1 [file Supplemental_Figures_Combined.docx]

**
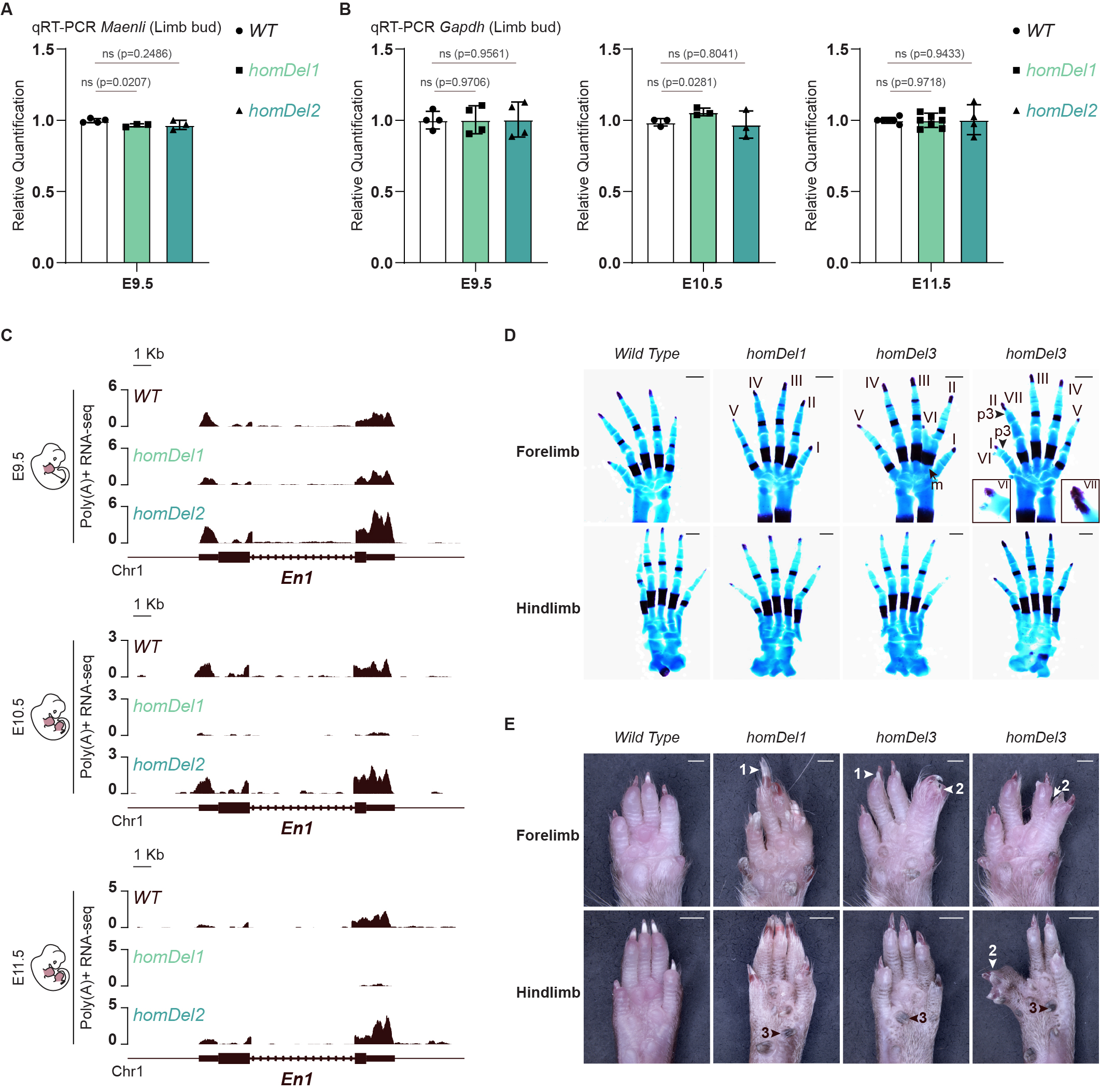
**

**Supplemental Fig. S1.** *homDel1* mouse model exhibits abnormal limb bud dorso-ventral patterning similar to *Maenli*^-/-^ and *En1* mutant mice. (A) Normalized qRT-PCRs of *Maenli* in E9.5 mouse limb embryos show that *homDel1* and *homDel2* did not significantly affect *Maenli* expression. Data were normalized to wild-type expression; one-tailed t-test; data are mean±SD; n = 4 *WT*, 3 *homDel1*, 3 *homDel2* at E9.5. ns, non-significant; p, p-value; WT, wild-type. (B) Normalized qRT-PCRs of *Gapdh* in E9.5, E10.5, and E11.5 mouse limb embryos show no significant changes in *Gapdh* expression upon *homDel1* and *homDel2* deletions. Data were normalized to wild-type expression; one-tailed t-test; data are mean±SD; n= 4 *WT*, 4 *homDel1*, 4 *homDel2* at E9.5; n= 3 *WT*, 3 *homDel1*, 3 *homDel2* at E10.5; n = 6 *WT*, 8 *homDel1*, 4 *homDel2* at E11.5. ns, non-significant; p, p-value; WT, wild-type. (C) Poly(A)^+^ RNA-seq profiles of E9.5, E10.5, and E11.5 mouse limb buds show that *homDel1* did not affect *En1* expression at E9.5 but resulted in an almost complete loss of *En1* expression at E10.5 and E11.5. By contrast, *homDel2* increased *En1* expression at E9.5, E10.5, and E11.5. WT, wild-type. n = 2 biologically independent *WT*, *homDel1*, and *homDel2* replicates. (D) Alcian blue (cartilage) and alizarin red (bone) stained limbs prepared from wild-type, *homDel1*, and *homDel3* E18.5 embryos. The *homDel1* mutant is indistinguishable from the wild-type. The *homDel3* mutant exhibits the presence of ectopic ventral digits (VI and VII) fused at the level of metacarpal or phalanges 3 in the forelimb. m, metacarpal; p, phalange. Scale bars, 500 µm; n = 10 *WT*, 8 *homDel1*, and 9 *homDel3*. (E) Ventral views of wild-type, *homDel1*, and *homDel3* adult (8-weeks) fore- and hind-paws illustrating the presence of ectopic ventral nails that manifest as circumferential nails or ventral nails formed opposite to the dorsal nails (1), and syndactyly (2). The pigmented metatarsal pads (3) are elongated and hardened, resembling nails. Scale bars, 1,000 µm for forelimbs and 2,000 µm for hindlimbs, respectively; n = 13 *homDel1* and 15 *homDel3*.

**
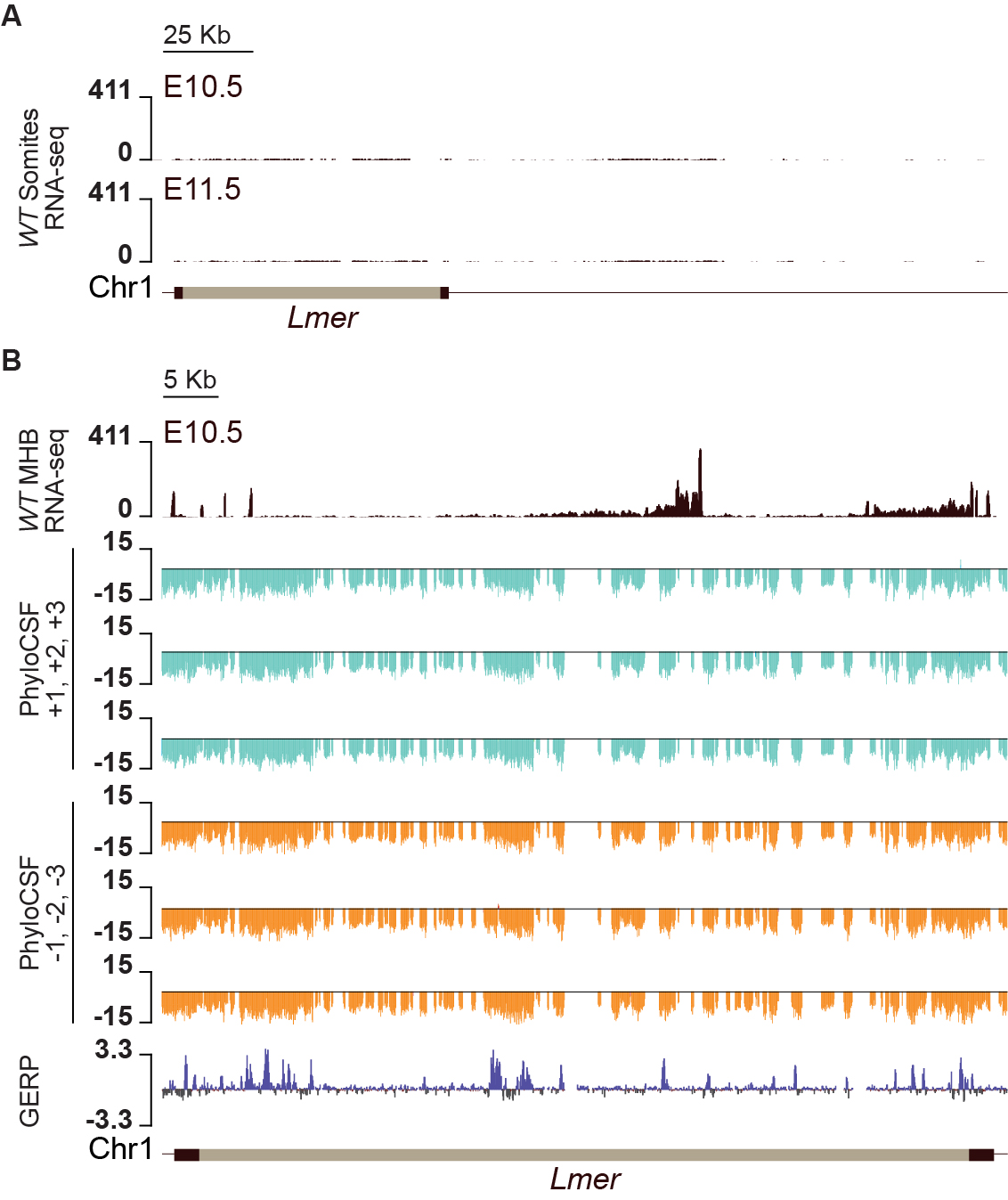
**

**Supplemental Fig. S2.** *Lmer* is expressed in the limb and midbrain/hindbrain boundary but not in the somites. (A) Poly(A)^+^ RNA-seq profiles of E10.5 and E11.5 wild-type (*WT*) mouse somites show no expression of *Lmer* in these embryonic structures. n = 2 biologically independent wild-type replicates. (B) Poly(A)^+^ RNA-seq profile of E10.5 wild-type (*WT*) mouse midbrain-hindbrain junction shows a Zoom in of the *Lmer* transcript (exon-intron composition). PhyloCSF tracks show a lack of protein coding potential for the *Lmer* transcript. Vertebrate sequence conservation tracks measured using the Genomic Evolutionary Rate Profiling (GERP) method show that the *Lmer* nucleotide sequence (exon composition) exhibits little conservation. n = 2 biologically independent wild-type poly(A)^+^ RNA-seq replicates.

**
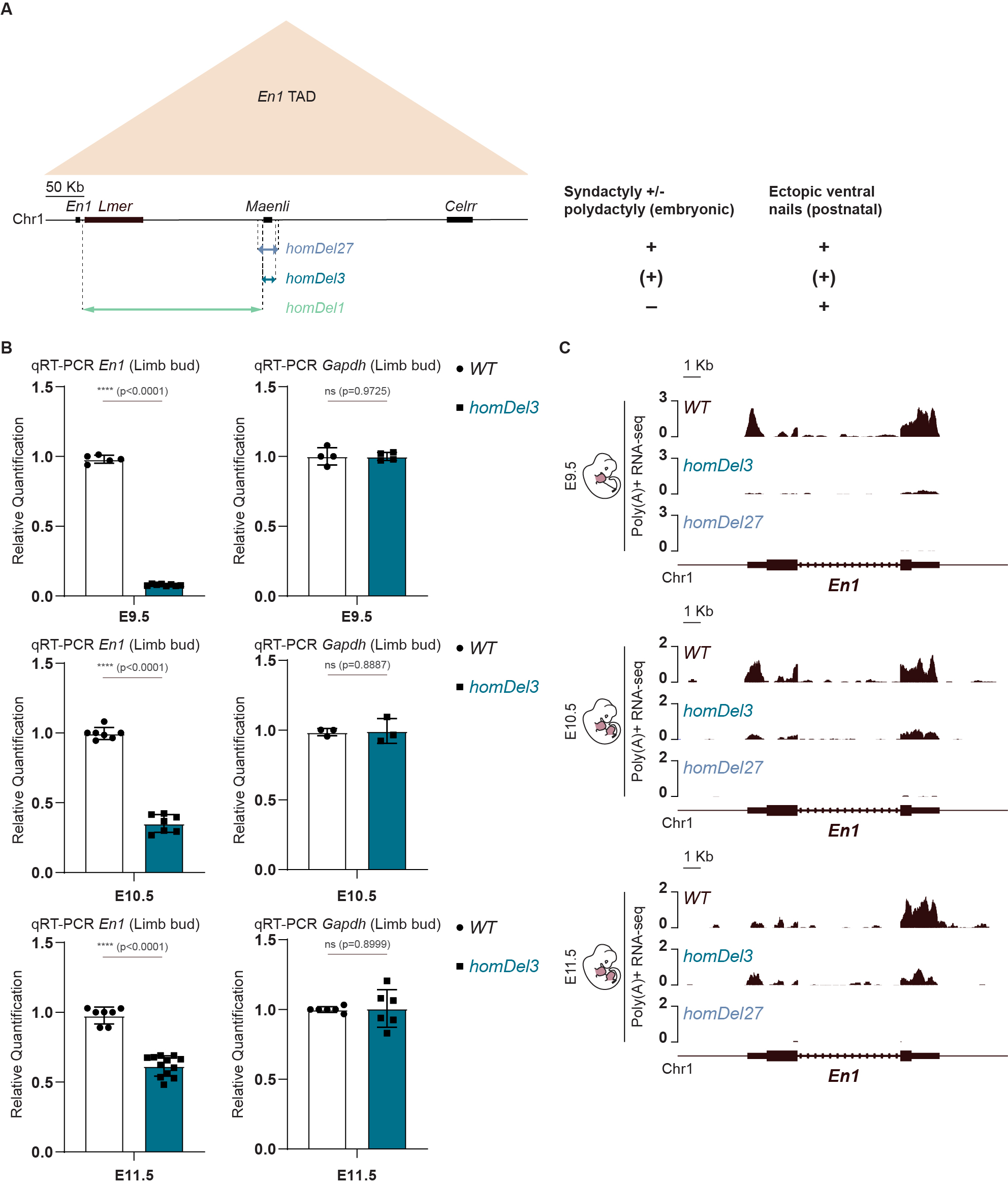
**

**Supplemental Fig. S3.** The overlapping genomic region between *homDel1* and *homDel27* contains the putative enhancers controlling *En1* expression in E10.5 and E11.5 mouse limb buds. (A) Schematic representation of the *En1* TAD (beige triangle) (chr1: 122,459,752-123,089,561 mm9) and the CRISPR-Cas9 genetic deletions affecting *En1* limb expression and causing abnormal limb bud dorsoventral patterning in mice. The main differential phenotypic (morphological) features of the mutants at E18.5 (syndactyly with or without polydactyly) and 8 weeks of age (ectopic ventral nails) are shown on the right. +, a syndactyly with or without polydactyly was observed in all forelimbs and ectopic ventral nails in all forelimb and hindlimb digits; (+), a syndactyly with or without polydactyly was observed in ~ 1 forelimb of each embryo and ectopic ventral nails in ~ 2-3 digits of each limb; -, the syndactyly and polydactyly were absent in all forelimbs. (B) Normalized qRT-PCRs of *En1* and *Gapdh* in E9.5, E10.5, and E11.5 mouse limb embryos show no significant changes in *Gapdh* expression upon deleting *Maenli* (*homDel3*). By contrast, this deletion significantly affected *En1* expression at E9.5, E10.5, and E11.5, with an almost complete loss at E9.5. *En1* expression was partially rescued at E10.5 and E11.5. Data were normalized to wild-type expression; one-tailed t-test; data are mean±SD; (n = 5 *WT*, 8 *homDel3* at E9.5; n = 7 *WT*, 7 *homDel3* at E10.5; n = 7 *WT*, 12 *homDel3* at E11.5 for *En1* expression quantification); (n = 4 *WT*, 4 *homDel3* at E9.5; n = 3 *WT*, 3 *homDel3* at E10.5; n = 6 *WT*, 6 *homDel3* at E11.5 for *Gapdh* expression quantification). ns, non-significant; *, (p < 0.01); p, p-value; WT, wild-type. (C) Poly(A)^+^ RNA-seq profiles of E9.5, E10.5, and E11.5 mouse limb buds show that *homDel27* caused a complete loss of *En1* expression at all three developmental stages. *homDel3* caused an almost complete loss of *En1* expression at E9.5 with a partial rescue at E10.5 and E11.5. WT, wild-type. n = 2 biologically independent *WT*, *homDel3*, and *homDel27* replicates.

**
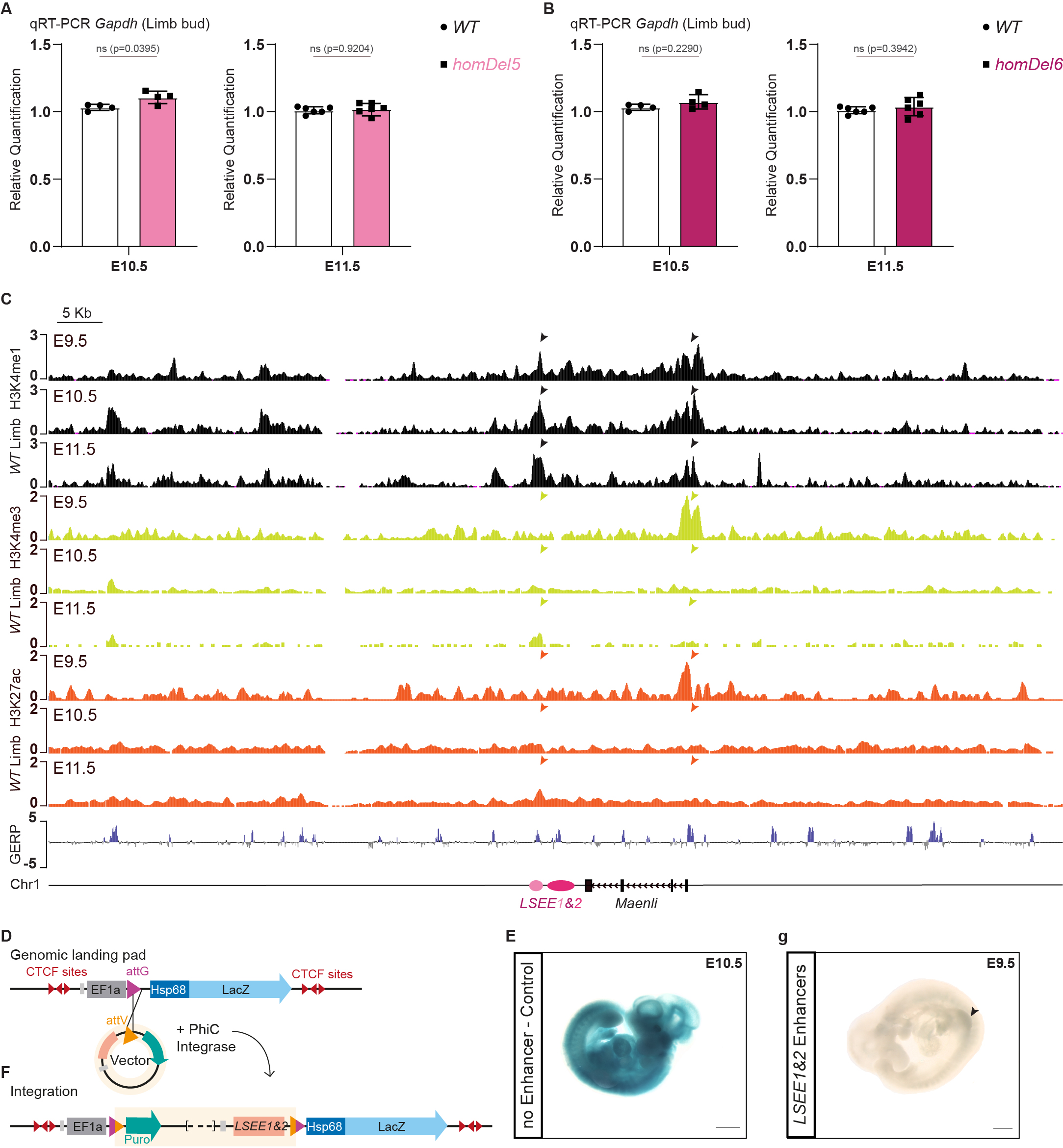
**

**Supplemental Fig. S4.** *In vivo* enhancer reporter assays test the enhancer and temporal activities of *LSEE1&2*. (A) Normalized qRT-PCRs of *Gapdh* in E10.5 and E11.5 mouse limb embryos show no significant changes in *Gapdh* expression upon deleting the single putative enhancer *LSEE1* (*homDel5*). Data were normalized to wild-type expression; one-tailed t-test; data are mean±SD; n = 4 *WT*, 4 *homDel5* at E10.5; n = 6 *WT*, 6 *homDel5* at E11.5. ns, non-significant; p, p-value; WT, wild-type. (B) Normalized qRT-PCRs of *Gapdh* in E10.5 and E11.5 mouse limb buds isolated from *homDel6* embryos show no significant changes in *Gapdh* expression at both developmental stages. Data were normalized to wild-type expression; one-tailed t-test; data are mean±SD; n = 4 *WT*, 4 *homDel6* at E10.5; n = 6 *WT*, 6 *homDel6* at E11.5. ns, non-significant; p, p-value; WT, wild-type. (C) H3K4me1, H3K4me3, and H3K27ac ChIP-seq profiles of E9.5, E10.5, and E11.5 wild-type (WT) mouse limb buds show the presence of the active epigenetic marks H3K4me3 and H3K27ac at the *Maenli* TSS at E9.5 but not at later stages; these active epigenetic marks are absent (E9.5 and E10.5) or detected at very low levels (E11.5) at *LSEE1&2*. H3K4me1 is detected at all developmental stages at the *Maenli* TSS and *LSEE1&2* CREs. The presence or absence of epigenetic marks is indicated using arrowheads. n = 2 biologically independent wild-type replicates. Vertebrate conservation is measured using the Genomic Evolutionary Rate Profiling (GERP) method. (D) Landing pad at the *Rosa26* locus and enhancer targeting plasmid. (E) Control experiment using integration of a non-enhancer plasmid integrated at the *Rosa26* locus. Scale bars, 1000 µm. (F) Genomic organization of the integrated enhancer-reporter construct at the *Rosa26* locus. (G) *LSEE1&2* regulatory elements do not drive limb-specific *LacZ* expression in E9.5 mouse limb embryos (indicated using an arrowhead). Scale bars, 500 µm.

**
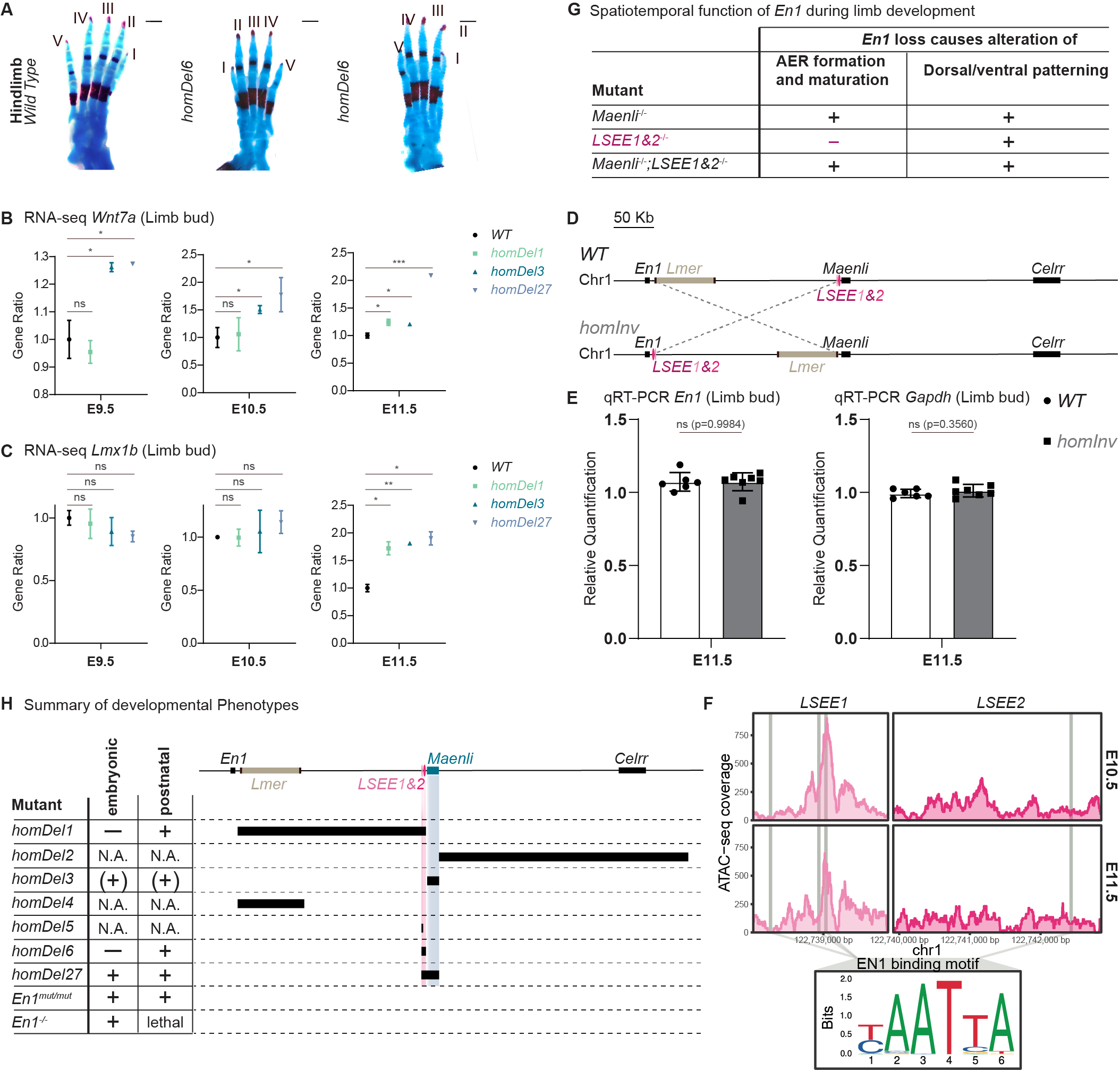
**

**Supplemental Fig. S5.** *En1* expression could be maintained through a positive feedback loop where EN1 binds to *LSEE1&*2. (A) Alcian blue (cartilage) and alizarin red (bone) stained limbs prepared from wild-type and *homDel6* E17.5 embryos. The *homDel6* mutants are indistinguishable from the wild-type. Scale bars, 500 µm; n = 4 *WT* and 8 *homDel6*. (B) Poly(A)^+^ RNA-seq profiles of E9.5, E10.5, and E11.5 mouse limb buds show that ectopic *Wnt7a* expression in the ventral limb ectoderm is significantly gained already at E9.5 in *homDel3* (*Maenli*^-/-^) and *homDel27* (*Maenli*^-/-^; *LSEE1&2*^-/-^) limb mutants, its expression is only gained from E11.5 in *homDel1* limb mutants (*LSEE1&2*^-/-^). WT, wild-type. n = 2 biologically independent *WT*, *homDel1*, *homDel3*, and *homDel27* replicates. ns, non-significant; *, (p < 0.05); p = 0.2529, 0.0174, 0.0151 at E9.5; p = 0.4165, 0.0331, 0.0460 at E10.5, p = 0.0205, 0.0187, 0.0005 at E11.5, respectively. (C) Poly(A)^+^ RNA-seq profiles of E9.5, E10.5, and E11.5 mouse limb buds show that ectopic *Lmx1b* expression in the ventral limb mesoderm is significantly gained from E11.5 in all limb mutants. WT, wild-type. n = 2 biologically independent *WT*, *homDel1*, *homDel3*, and *homDel27* replicates. ns, non-significant; *, (p < 0.05); p = 0.3324, 0.1718, 0.0503 at E9.5; p = 0.4714, 0.3700, 0.1004 at E10.5; p = 0.0086, 0.0017, 0.0056 at E11.5. (D) Schematic representation of the CRISPR-Cas9 genetic inversion repositioning the *LSEE1&2* enhancers away from *Maenli* within the *En1* regulatory landscape (*homInv*). (E) Normalized qRT-PCRs of *En1* and *Gapdh* in E11.5 mouse limb embryos show no significant changes in *En1* and *Gapdh* expression upon repositioning the *LSEE1&2* enhancers away from *Maenli* (*homInv*). Data were normalized to wild-type expression; one-tailed t-test; data are mean±SD; n= 6 *WT*, 7 *homInv*. ns, non-significant; p, p-value; WT, wild-type. (F) The search for EN1 transcription factor binding sites within *LSEE1&2*, using the reported JASPAR binding motif (Rauluseviciute et al. 2024), identifies four conserved potential EN1 binding sites. Two of the conserved EN1 binding motifs are located in the genomic region which is enriched in aligned ATAC reads within *LSEE1*. (G) Table summarizing the phenotypes observed for *Maenli*^-/-^, *LSEE1&2*^-/-^, and (*Maenli*^-/-^;*LSEE1&2*^-/-^), indicating distinct essential spatiotemporal roles of *En1* during limb development and patterning. +, presence; -, absence. (H) Schematic representation of the CRISPR-Cas9 genetic deletions generated in this study and previously reported (hom*Del27*) (Allou et al. 2021) is shown. Table summarizing and showing differences in the embryonic and postnatal phenotypes according to the different mutations is also shown. The phenotypes of *homDel27*, *En1*^mut/mut^, and *En1*^-/-^ mice have been described in previous studies (Wurst et al. 1994; Loomis et al. 1996; Hanks et al. 1998; Allou et al. 2021; Ma et al. 2024). +, a syndactyly with or without polydactyly was observed in all forelimbs and ectopic ventral nails in all forelimb and hindlimb digits; (+), a syndactyly with or without polydactyly was observed in ~ 1 forelimb of each embryo and ectopic ventral nails in ~ 2-3 digits of each limb; -, the syndactyly and polydactyly were absent in all forelimbs; N.A., not assessed.
