# Supplementary material for: Temporal loss of En1 during limb development causes distinct phenotypes
Source: Genes Dev. 2026 May 1;40(9-10):627–37. doi: 10.1101/gad.353542.125 (PMC13138323; doi:10.1101/gad.353542.125)
Supplement: Supplement 2 [file Supplemental_Information.docx]

**SUPPLEMENTAL INFORMATION**

**Temporal loss of *En1* during limb development causes distinct phenotypes** (Main Title)

***Maenli* and *LSEE1&2* coordinate temporal limb patterning** (Running Title)

**Authors:** Alessa R. Ringel^1,2^, Natalia Benetti^1^, Andreas Magg^1,2,3^, Fabian Groll^4,5^, Robert Schöpflin^1,6^, Mira Kühnlein^1^, Asita Carola Stiege^1^, Ute Fischer^1^, Lars Wittler^7^, Laurence Game^4,5^, Stephan Lorenz^8^, George Young^4,5^, Stefan Mundlos^1,2,3, #^ & Lila Allou^1,4,5, #^

**Affiliations:** ^1^RG Development and Disease, Max-Planck Institute for Molecular Genetics, Berlin, Germany; ^2^Institute for Medical and Human Genetics, Charité-Universitätsmedizin Berlin, Berlin, Germany; ^3^Berlin-Brandenburg Center for Regenerative Therapies, Charité-Universitätsmedizin Berlin, Berlin, Germany; ^4^Genomic Variation and Disease Research Group, Medical Research Council Laboratory of Medical Sciences (MRC LMS), London, UK; ^5^Institute of Clinical Sciences, Faculty of Medicine, Imperial College London, London, UK; ^6^Department of Computational Molecular Biology, Max-Planck Institute for Molecular Genetics, Berlin, Germany; ^7^Department of Developmental Genetics, Max-Planck Institute for Molecular Genetics, Berlin, Germany; ^8^Sequencing Core Facility, Max-Planck Institute for Molecular Genetics, Berlin, Germany; ^#^ Author for correspondence

(mundlos@molgen.mpg.de; l.allou@lms.mrc.ac.uk)

**This file includes:**

Supplemental Methods

Supplemental Methods References

Supplemental Data Captions

**Supplemental Methods**

**RNA isolation and quantitative reverse transcription PCR (qRT–PCR).** To quantify RNA levels in wild-type and mutant mice at E9.5, E10.5, and E11.5 developmental stages (G4 background), limb buds were micro-dissected in cold PBS, immediately snap-frozen, and stored at −80 °C. Total RNAs were extracted using the RNeasy Mini Kit (QIAGEN) according to the manufacturer’s instructions. RNAs were treated with DNase I (Thermo Fisher Scientific) at 37 °C for 15 min followed by 10 min incubation at 70 °C for DNase I inactivation. Complementary DNAs (cDNAs) were generated using the Superscript III First-Strand Synthesis System (Thermo Fisher Scientific) whereby 1 µg of RNA was reverse-transcribed using oligo(dT)20. To quantify the relative abundance of transcripts, qRT–PCR analyses were done in technical triplicates using the Power Sybr Green Master Mix (Applied Biosystems) as described above. The dosage of each amplicon was normalized to the endogenous control amplicon (*Rps9* cDNAs). The 2^-ΔΔ^*^CT^* method (Livak and Schmittgen 2001) was used to calculate the fold change between wild-type and mutant samples. Using a one-tailed Student’s *t*-test, we tested the ability of mutations to result in changes in *Gapdh*, *Maenli* and/or *En1* gene expression. The variance in mutant and wild-type samples was assumed equal. The sequences of all qRT-PCR primers used in this study are listed in **Supplemental Table S1**.

**Skeletal preparation.** E17.5/ E18.5 fetuses (G4 background) were kept in H2O for 1–2 h at RT and heat-shocked at 65 °C for 1 min. The skin was taken off and the abdominal and thoracic viscera were removed using forceps. The fetuses were then fixed in 100% ethanol overnight. Afterwards, the cartilage was stained overnight using alcian blue staining solution (150 mg/l alcian blue 8GX in 80% ethanol and 20% acetic acid). Fetuses were then rinsed and post-fixed in 100% ethanol overnight. After 24 h, initial clearing was done by incubating the fetuses for 20 min in 1% potassium hydroxide in H2O, followed by alizarin red (50 mg/l alizarin red S in 0.2% potassium hydroxide) staining of bones overnight. Following this, rinsing and clearing was done for several days using low concentrations of potassium hydroxide. The stained embryos were dissected in 80% glycerol and limbs were imaged using a ZEISS SteREO Discovery.V12 with cold light source CL9000 microscope and Leica DFC420 digital camera.

**Phenotypic evaluation.** Phenotypic analyses for 6-8 weeks mutant mouse lines were carried out for at least 10 animals per analysis.

**SureSelect design.** The capture Hi-C SureSelect library probes were designed over the genomic interval (mm9, chr1: 119,650,000–124,400,000) using the SureDesign online tool from Agilent.

**Capture Hi-C (cHi-C).** cHi-C libraries were prepared from E10.5 wild-type limb buds (CD1 background). cHi-C experiments were performed as duplicates. Per biological replicate, 10 pairs E10.5 limb buds (2~3 × 10^6^ cells) were micro-dissected in PBS at RT. A single-cell suspension was obtained by incubating the tissue for 10 min at 37 °C in 1 ml Gibco trypsin-EDTA 0.05% (Thermo Fisher Scientific). Cells were resuspended in 5 ml 10% fetal bovine serum/PBS and fixed by adding 5 ml 4% formaldehyde (Sigma-Aldrich) at a final concentration of 2%. Cells were mixed for 10 min at RT. Fixation was quenched using 1.425 M glycine (Merck) on ice and immediately centrifuged at 2100 r.p.m. for 8 min. Supernatant was removed and the pellet resuspended in lysis buffer (final concentration of 10 mM Tris, pH 7.5, 10 mM NaCl, 5 mM MgCl2, 0.1 M EDTA, and 1× cOmplete protease inhibitors (Sigma-Aldrich)) and incubated on ice for 10 min. Cells were then centrifuged at 2900 r.p.m. for 5 min at 4 °C, followed by removal of supernatant, snap-freezing, and storage at −80 °C. For the preparation of the 3C library, the pellet was resuspended in 60 µl 10× DpnII buffer (Thermo Fisher Scientific), and incubated with 15 µl 10% SDS for 1 h at 37 °C and 900 r.p.m. 150 µl 10% Triton X-100 was then added and the pellet was incubated for 1 h at 37 °C and 900 r.p.m. 600 µl of 1× DpnII buffer was added to the samples. A 10-µl aliquot was taken as undigested control and stored at −20 °C. The chromatin was digested using 40 µl 10 U/µl DpnII for 4 h at 37 °C and 900 r.p.m.; another 20 µl of DpnII enzyme was then added and samples were incubated overnight at 37 °C with rotation. After overnight incubation, samples were supplemented with 20 µl DpnII enzyme and incubated for four more hours at 37 °C with rotation. The DpnII restriction enzyme was inactivated at 65 °C for 20 min. Next, the digested chromatin was diluted and religated in 5.1 ml H2O, 700 µl 10× ligation buffer (Thermo Fisher Scientific), and 1.67 µl 30 U/µl T4 DNA ligase (Thermo Fisher Scientific). Ligation reactions were incubated at 16 °C overnight with rotation. A 100-µl aliquot was taken to test ligation efficiency and stored at −20 °C. The chimeric chromatin products and test aliquots were de-cross-linked overnight by adding 30 µl and 5 µl 20 mg/ml proteinase K, respectively, and incubated at 65 °C overnight. Following this, 30 µl or 5 µl 10 mg/ml RNase A was added to the samples and aliquots, respectively, and incubated for 45 min at 37 °C. Chromatin was then precipitated by adding 1 volume phenol-chloroform to the samples and aliquots, vigorously shaking them, followed by centrifugation at 3,750 r.p.m. at RT for 15 min. The upper phase containing the chromatin was transferred to a new tube and the volume was adjusted to 7 ml with H2O. Samples were then supplemented with 1 ml 3M NaAc, pH 5.6, 35 ml 100% ethanol, and 7 µl 20 mg/ml glycogen and frozen overnight at −80 °C. The precipitated chromatin was isolated by centrifugation at 8,350 g for 20 min at 4 °C. The chromatin pellet was washed with 10 ml 70% ethanol and further centrifuged at 3,300 g for 15 min at 4 °C. Finally, the 3C library chromatin pellet was dried at RT and resuspended in 150 µl 10 mM Tris-HCl, pH 7.5 at 37 °C. To check the 3C library, the undigested, digested, and ligated aliquots were loaded on a 1% agarose gel. The 3C library was then sheared using a Covaris sonicator (duty cycle: 10%; intensity: 5; cycles per burst: 200; time: 6 cycles of 60 s each; set mode: frequency sweeping; temperature: 4-7 °C). Adaptors were added to the sheared DNA and amplified according to the manufacturer’s instructions for Illumina sequencing (Agilent). The library was hybridized to the custom-designed SureSelect beads and indexed for sequencing (150 bp paired-end) following the manufacturer’s instructions (Agilent).

**cHi-C processing.** FASTQ files were processed with the HiCUP pipeline v0.6.1 (Wingett et al. 2015) (no size selection, Nofill: 1, Format: Sanger) using Bowtie2 v2.3.4.1 (Langmead and Salzberg 2012) for mapping short reads to the reference genome mm9. After the mapping, filtering and deduplication steps of the HiCUP pipeline, replicates were merged by combining their bam files. Juicer tools v1.7.6 (Durand et al. 2016) was used to generate binned contact maps and to normalize maps by Knights and Ruiz (KR) matrix balancing (Rao et al. 2014). For the generation of the contact maps, only the genomic region (chr1:120,200,001-124,400,000, mm9), part of the region enriched in the capturing step, was considered. Therefore, only read-pairs mapping to the region of interest were kept and their coordinates were shifted towards the origin by the offset of this region. Afterwards, cHi-C maps were generated with the Juicer tools ‘pre’ command using a custom chrom.sizes file containing only the length of the genomic region of interest (4.2 Mb) and applying a minimum MAPQ of 30. After exporting KR normalized cHi-C maps at 10kb bin size, coordinates were shifted back to mm9 coordinates. cHi-C maps were displayed as heatmaps in which values above the 98.5^th^ percentile were truncated to improve visualization.

**Virtual 4C profile generation.** In order to obtain more fine-grained interaction profile for the *En1* promoter, a virtual 4C-like profile was generated from mapped, filtered and deduplicated cHi-C read pairs obtained from the HiCUP pipeline. A read-pair was considered in the profile, when it had a MAPQ≥30 and one read mapped to the defined viewpoint region while the other one mapped outside of it. Out of these pairs, reads mapping outside of the viewpoint region were counted per restriction fragment and binned to a 1kb grid. The count value of restriction fragments spanning more than one bin was distributed proportionally to the corresponding bins. Afterwards, the profile was smoothed by averaging over five bins and scaled by the factor 10^3^ / counts within the enriched region. For the computation of the scaling factor, the viewpoint region and a margin ±5 kb around it were excluded. The processing was performed with custom Java code using htsjdk v.2.12.0 (<https://samtools.github.io/htsjdk/>).

**RNA-sequencing (RNA-seq).** E9.5 forelimb buds, and E10.5 and E11.5 limb buds were micro-dissected from wild-type and mutant embryos (G4 background) in cold PBS, immediately snap-frozen, and stored at -80 °C. RNA-seq experiments were performed in duplicates. Total RNAs were extracted using the RNeasy Mini Kit (QIAGEN) according to the manufacturer’s instructions. RNAs were treated with DNase I (Thermo Fisher Scientific) at 37 °C for 15 min followed by 10 min incubation at 70 °C for DNase I inactivation. Samples were poly-A enriched and sequenced (150 pb paired-end) using Illumina technology following standard protocols.

**RNA-seq data processing.** Raw FASTQ reads were trimmed with fastp 0.23.3 (Chen et al. 2018) using the following options: ‘-3 --cut_tail_window_size 1 --cut_tail_mean_quality 20 --detect_adaptor_for_pe --correction --trim_poly_x --poly_x_min_length 5’. Transcript expression was quantified with salmon 1.10.2 (Patro et al. 2017) using an index built with the ‘-k 31 –keepDuplicates’ options on the GRCm37 transcriptome (Ensembl release 54) and the whole genome as decoys. Quantification was done with the ‘-l A --seqBias --gcBias --posBias --numBootstraps 30’ options before performing differential expression analysis in R with DESeq2 1.40.2. The results were visualized using ggplot2 (Wickham 2016).

**ATAC-seq data processing.** Raw sequencing files were processed with Cutadapt to trim the adapter sequence (Martin 2011), Bowtie2 was used for mapping (Langmead and Salzberg 2012). SAMtools was used for filtering, sorting, and duplicate removal (Li et al. 2009). deepTools was used to create coverage tracks (Ramirez et al. 2016). Calling of peaks was performed using Genrich with default settings ([https://github.com/jsh58/Genrich)](https://github.com/jsh58/Genrich)51) (Gaspar 2018).

**ChIP-sequencing (ChIP-seq).** Chromatin Immunoprecipitation was performed as previously described (Ibrahim et al. 2013). ChIP-seq experiments were performed in duplicates. Briefly, 80-100 pairs E9.5 forelimbs (CD1 background) were micro-dissected in PBS at RT. A single-cell suspension was obtained by incubating the tissue for 10 min at 37 °C in 1 ml Gibco trypsin-EDTA 0.05% (Thermo Fisher Scientific). Cells were resuspended in 15 ml 10% fetal bovine serum/PBS and fixed by adding 5 ml 4% formaldehyde (Sigma-Aldrich) at a final concentration of 1%. Cells were mixed for 10 min at 4 °C. Fixation was quenched using 1.425 M glycine (Merck) on ice and immediately centrifuged at 2100 r.p.m. for 8 min. Supernatant was removed and the pellet was immediately snap-frozen and stored at -80 °C. The extraction of nuclear lysate was performed as described in Lee *et al.* (Lee et al. 2006) and chromatin was sonicated with a Diagenode Bioruptor (45 cycles with 30 sec pulse, 30 sec pause, HI power). For ChIP, 25–35 μg of chromatin was incubated with 6–8 μg of antibody (anti-H3K4me1, Diagenode, cat. No. C15410037) overnight. Following this, blocked magnetic beads were added to the chromatin/ antibody mixture and incubated overnight. Next, samples were washed 6 times with RIPA buffer and one time with TE buffer (Lee et al. 2006). After elution, the preparation of the library for pulled down DNA was performed as previously described (Ibrahim et al. 2013). ChIP-seq libraries were sequenced (75 bp single-end) using Illumina technology following standard protocols.

**ChIP-seq data processing.** Single-end ChIP-seq reads were mapped with bowtie (v2.2.6) (Langmead and Salzberg 2012) to mm9, filtered for mapping quality MAPQ ≥ 10, and duplicates were removed using samtools rmdup (v1.8). For generating coverage tracks, reads were extended to 300 bp and scaled to r.p.m. (10^6^ per number of unique reads) using bedtools genomecov v2.27.1.

**Whole-mount *in situ* hybridization (WISH).** RNA expression in E11.5/E12.5 mouse embryos (G4 and C57BL/6 backgrounds) was assessed by WISH using a digoxigenin-labeled antisense riboprobe transcribed from a cloned probe (PCR DIG Probe Synthesis Kit, Roche). Whole embryos were fixed overnight in 4% PFA/PBS. The embryos were then washed in PBS-Tween (PBST, 0.1% Tween), dehydrated for 10 minutes (min) each in 25%, 50%, and 75% methanol/PBST, and finally stored at −20 °C in 100% methanol. The WISH protocol was as follows. Embryos were rehydrated on ice in reverse methanol/PBST steps, washed in PBST, bleached in 6% H2O2/PBST for 1 h and washed again in PBST. Embryos were then treated or not (embryos probed with *Lmx1b* were treated while embryos probed with *Fgf8* and *Wnt7a* were not) in 10 µg/ml proteinase K/PBST for 3 min, incubated in glycine/PBST, washed in PBST, and finally refixed for 20 min with 4% PFA/PBS, 0.2% glutaraldehyde, and 0.1% Tween 20. After further washing steps with PBST, embryos were incubated at 68 °C in L1 buffer (50% deionized formamide, 5× saline sodium citrate, 1% SDS, 0.1% Tween 20 in diethyl pyrocarbonate, pH 4.5) for 10 min. Embryos were then incubated for 2 h at 68 °C in hybridization buffer 1 (L1 with 0.1% transfer RNA and 0.05% heparin). Afterwards, embryos were incubated overnight at 68 °C in hybridization buffer 2 (hybridization buffer 1 with 0.1% transfer RNA and 0.05% heparin and 1/500 digoxigenin-probe). After overnight hybridization, removal of the unbound probe was done through a series of washing steps 3 × 30 min each at 68 °C: L1; L2 (50% deionized formamide, 2× saline sodium citrate pH 4.5, 0.1% Tween 20 in diethyl pyrocarbonate, pH 4.5); and L3 (2× saline sodium citrate pH 4.5, 0.1% Tween 20 in diethyl pyrocarbonate, pH 4.5). Subsequently, embryos were treated for 1 h with RNase solution (0.1 M NaCl, 0.01 M Tris pH 7.5, 0.2% Tween 20, 100 µg/ml RNase A in H2O), followed by washing in Tris-buffered saline, 0.1% Tween 20 (TBST 1) (140 mM NaCl, 2.7 mM KCl, 25 mM Tris-HCl, 1% Tween 20, pH 7.5). Embryos were then blocked for 2 h at room temperature (RT) in blocking solution (TBST 1 with 2% fetal bovine serum and 0.2% bovine serum albumin (BSA)), followed by incubation at 4 °C overnight in blocking solution containing 1/5,000 anti-digoxigenin-alkaline phosphatase. After overnight incubation, removal of unbound antibody was done through a series of washing steps 8 × 30 min at RT with TBST 2 (TBST with 0.1% Tween 20 and 0.05% levamisole/tetramisole) and left overnight at 4 °C. 24 h later, staining of the embryos was initiated by washing at RT with alkaline phosphatase buffer (0.02 M NaCl, 0.05 M MgCl2, 0.1% Tween 20, 0.1 M Tris-HCl, and 0.05% levamisole/tetramisole in H2O) 3 × 20 min, followed by staining with BM Purple AP Substrate (Roche). At least three embryos were analyzed from each mutant genotype. The limb buds of stained embryos were imaged using a ZEISS SteREO Discovery.V12 with cold light source CL9000 microscope and Leica DFC420 digital camera. The sequences of primers used to generate *Lmx1b* and *Wnt7a* WISH probes for this study are listed in **Supplemental Table S1**. The *Fgf8* probe was previously used in *Liska et al* (Liska et al. 2010).

**Generation of Landing Pad mESC Line**: The landing pad mESC line was generated as previously described (Phan et al. 2025). Briefly, cells were co-transfected with 8 µg of the knock-in (KI) construct SYN102_LP_R26_CTCF_attG_Array_Hsp_LacZ and 8 µg of the sgRNA plasmid (pSpCas9(BB)-2A-Puro (PX459) V2.0 (Addgene Plasmid #62988)) using FuGENE HD Transfection Reagent (Promega), following the manufacturer’s protocol. The knock-in construct consisted of homology arms (HA) (5’ HA / mm10 / chr6:113,076,061–113,078,081; 3’ HA / mm10 / chr6:113,075,226–113,076,060), an array of CTCF sites, the EF1α promoter, and an array of attG sites, including a site that is recognized by PhiC integrase (attB sequences of LSR, Bxb1, Pa03, Kp03, PhiC sequence taken from (Durrant et al. 2023); EF1α sequence taken from EF1a mCherry Addgene Plasmid #129431). The full sequence of the plasmid is provided as a Fasta file (**Supplemental Fasta File S1**). The sequence of sgRNA used for CRISPR targeting and primers used for genotyping of the ESC clones are listed in **Supplemental Table S1**.

**EN1 binding motif search in LSEE1&2 sequences.** The binding site motif for EN1 (MA0027.3) was downloaded from the JASPAR database (Rauluseviciute et al. 2024) and motif occurrences in the sequences of *LSEE1* and *LSEE2* were detected with MOODS 1.9.4.2 (Korhonen et al. 2017), using a significance cutoff of ‘-p 0.0005’. The results were visualized in R using ggplot2 (Wickham 2016).

**Supplemental Methods References**

Chen S, Zhou Y, Chen Y, Gu J. 2018. fastp: an ultra-fast all-in-one FASTQ preprocessor. *Bioinformatics* **34**: i884-i890.

Durand NC, Shamim MS, Machol I, Rao SS, Huntley MH, Lander ES, Aiden EL. 2016. Juicer Provides a One-Click System for Analyzing Loop-Resolution Hi-C Experiments. *Cell Syst* **3**: 95-98.

Durrant MG, Fanton A, Tycko J, Hinks M, Chandrasekaran SS, Perry NT, Schaepe J, Du PP, Lotfy P, Bassik MC et al. 2023. Systematic discovery of recombinases for efficient integration of large DNA sequences into the human genome. *Nat Biotechnol* **41**: 488-499.

Gaspar J. 2018. Genrich: Detecting sites of genomic enrichment

Ibrahim DM, Hansen P, Rodelsperger C, Stiege AC, Doelken SC, Horn D, Jager M, Janetzki C, Krawitz P, Leschik G et al. 2013. Distinct global shifts in genomic binding profiles of limb malformation-associated HOXD13 mutations. *Genome Res* **23**: 2091-2102.

Korhonen JH, Palin K, Taipale J, Ukkonen E. 2017. Fast motif matching revisited: high-order PWMs, SNPs and indels. *Bioinformatics* **33**: 514-521.

Langmead B, Salzberg SL. 2012. Fast gapped-read alignment with Bowtie 2. *Nat Methods* **9**: 357-359.

Lee TI, Johnstone SE, Young RA. 2006. Chromatin immunoprecipitation and microarray-based analysis of protein location. *Nat Protoc* **1**: 729-748.

Li H, Handsaker B, Wysoker A, Fennell T, Ruan J, Homer N, Marth G, Abecasis G, Durbin R, Genome Project Data Processing S. 2009. The Sequence Alignment/Map format and SAMtools. *Bioinformatics* **25**: 2078-2079.

Liska F, Snajdr P, Stricker S, Gosele C, Krenova D, Mundlos S, Hubner N. 2010. Impairment of Sox9 expression in limb buds of rats homozygous for hypodactyly mutation. *Folia Biol (Praha)* **56**: 58-65.

Livak KJ, Schmittgen TD. 2001. Analysis of relative gene expression data using real-time quantitative PCR and the 2(-Delta Delta C(T)) Method. *Methods* **25**: 402-408.

Martin M. 2011. Cutadapt removes adapter sequences from high-throughput sequencing reads. *EMBnetjournal; Vol 17, No 1: Next Generation Sequencing Data AnalysisDO - 1014806/ej171200*.

Patro R, Duggal G, Love MI, Irizarry RA, Kingsford C. 2017. Salmon provides fast and bias-aware quantification of transcript expression. *Nat Methods* **14**: 417-419.

Phan MHQ, Zehnder T, Puntieri F, Magg A, Majchrzycka B, Antonovic M, Wieler H, Lo BW, Baranasic D, Lenhard B et al. 2025. Conservation of regulatory elements with highly diverged sequences across large evolutionary distances. *Nat Genet* **57**: 1524-1534.

Ramirez F, Ryan DP, Gruning B, Bhardwaj V, Kilpert F, Richter AS, Heyne S, Dundar F, Manke T. 2016. deepTools2: a next generation web server for deep-sequencing data analysis. *Nucleic Acids Res* **44**: W160-165.

Rao SS, Huntley MH, Durand NC, Stamenova EK, Bochkov ID, Robinson JT, Sanborn AL, Machol I, Omer AD, Lander ES et al. 2014. A 3D map of the human genome at kilobase resolution reveals principles of chromatin looping. *Cell* **159**: 1665-1680.

Rauluseviciute I, Riudavets-Puig R, Blanc-Mathieu R, Castro-Mondragon JA, Ferenc K, Kumar V, Lemma RB, Lucas J, Cheneby J, Baranasic D et al. 2024. JASPAR 2024: 20th anniversary of the open-access database of transcription factor binding profiles. *Nucleic Acids Res* **52**: D174-D182.

Wickham H. 2016. Data Analysis. in *ggplot2: Elegant Graphics for Data Analysis* (ed. H Wickham), pp. 189-201. Springer International Publishing, Cham.

Wingett S, Ewels P, Furlan-Magaril M, Nagano T, Schoenfelder S, Fraser P, Andrews S. 2015. HiCUP: pipeline for mapping and processing Hi-C data. *F1000Res* **4**: 1310.

**Supplemental Data Captions**

**Supplemental Table S1**: List of primer sequences and sgRNAs for CRISPR targeting, WISH, and enhancer reporter assays.

**Supplemental Table S2**: Sequencing breakpoints of the homozygous deletions and inversion, and genomic coordinates of *Maenli* and *Lmer*.

**Supplemental Fasta File S1**: Full sequence of the plasmid SYN102_LP_R26_CTCF_attG_Array_Hsp_LacZ.

**Supplemental Fasta File S2**: Full sequence of the plasmid SYN72-*LSEE1&2*.
